# Grouping of orally ingested silica nanomaterials via use of an integrated approach to testing and assessment to streamline risk assessment

**DOI:** 10.1186/s12989-022-00508-4

**Published:** 2022-12-02

**Authors:** Luisana Di Cristo, Victor C. Ude, Georgia Tsiliki, Giuseppina Tatulli, Alessio Romaldini, Fiona Murphy, Wendel Wohlleben, Agnes G. Oomen, Pier P. Pompa, Josje Arts, Vicki Stone, Stefania Sabella

**Affiliations:** 1 D3 PharmaChemistry, Nanoregulatory Group, Italian Institute of Technology, Via Morego, 30, 16163 Genoa, Italy; 2 Nano Safety Research Group, School of Engineering and Physical Sciences, Heriot Watt University, Edinburgh, EH14 4AS UK; 3 Institute for the Management of Information Systems, Athena Research Center, Marousi, Greece; 4 Nanobiointeractions & Nanodiagnostics, Istituto Italiano Di Tecnologia (IIT), Via Morego, 30, 16163 Genoa, Italy; 5 Department Material Physics and Department of Experimental Toxicology & Ecology, BASF SE, Ludwigshafen, Germany; 6 National Institute for Public Health and the Environment (RIVM), Bilthoven, The Netherlands; 7 Institute for Biodiversity and Ecosystem Dynamics, University of Amsterdam, Amsterdam, The Netherlands; 8 Nouryon, Arnhem, The Netherlands

**Keywords:** Oral dissolution, Nanoforms, Ingestion, Local toxicity, Systemic toxicity, Similarity assessment

## Abstract

**Background:**

Nanomaterials can exist in different nanoforms (NFs). Their grouping may be supported by the formulation of hypotheses which can be interrogated via integrated approaches to testing and assessment (IATA). IATAs are decision trees that guide the user through tiered testing strategies (TTS) to collect the required evidence needed to accept or reject a grouping hypothesis. In the present paper, we investigated the applicability of IATAs for ingested NFs using a case study that includes different silicon dioxide, SiO_2_ NFs. Two oral grouping hypotheses addressing local and systemic toxicity were identified relevant for the grouping of these NFs and verified through the application of oral IATAs. Following different Tier 1 and/or Tier 2 in vitro methods of the TTS (i.e., in vitro dissolution, barrier integrity and inflammation assays), we generated the NF datasets. Furthermore, similarity algorithms (e.g., Bayesian method and Cluster analysis) were utilized to identify similarities among the NFs and establish a provisional group(s). The grouping based on Tier 1 and/or Tier 2 testing was analyzed in relation to available Tier 3 in vivo data in order to verify if the read-across was possible and therefore support a grouping decision.

**Results:**

The measurement of the dissolution rate of the silica NFs in the oro-gastrointestinal tract and in the lysosome identified them as gradually dissolving and biopersistent NFs. For the local toxicity to intestinal epithelium (e.g. cytotoxicity, membrane integrity and inflammation), the biological results of the gastrointestinal tract models indicate that all of the silica NFs were similar with respect to the lack of local toxicity and, therefore, belong to the same group; in *vivo* data (although limited) confirmed the lack of local toxicity of NFs. For systemic toxicity, Tier 1 data did not identify similarity across the NFs, with results across different decision nodes being inconsistent in providing homogeneous group(s). Moreover, the available Tier 3 in vivo data were also insufficient to support decisions based upon the obtained in vitro results and relating to the toxicity of the tested NFs.

**Conclusions:**

The information generated by the tested oral IATAs can be effectively used for similarity assessment to support a grouping decision upon the application of a hypothesis related to toxicity in the gastrointestinal tract. The IATAs facilitated a structured data analysis and, by means of the expert’s interpretation, supported read-across with the available in vivo data. The IATAs also supported the users in decision making, for example, reducing the testing when the grouping was well supported by the evidence and/or moving forward to advanced testing (e.g., the use of more suitable cellular models or chronic exposure) to improve the confidence level of the data and obtain more focused information.

**Supplementary Information:**

The online version contains supplementary material available at 10.1186/s12989-022-00508-4.

## Background

European regulations for chemicals encourage the use of grouping and read-across approaches to reduce the need to test the hazard of a variety of nanoforms (NFs) of nanomaterials (NMs), on a case-by-case basis [1, 2]. The project GRACIOUS (funded by the European Commission), has generated a framework to streamline the implementation of grouping and read-across for NFs to integrate the industrial and regulatory grouping concepts (both human and environmental) [3]. In depth analyses of events in the life cycle of NFs and the biological pathways, which can influence their potential toxicity were collected in order to define a methodology and generate the GRACIOUS Grouping and Read-Across Framework. As no standardized way to generate a hypothesis for grouping exists, a template was designed to guide the user to structure and integrate the important elements required to generate a hypothesis suitable for grouping [3, 4]. The template includes sections which provide details on: (i) the purpose and the context for using the grouping hypothesis, (ii) the ‘life cycle’ of the NF (*e.g*., exposure scenario(s)), (iii) the descriptions of ‘What they are’ (physicochemical characteristics) ‘Where they go’ (environmental fate and behavior, uptake and toxicokinetics), ‘What they do’ ((eco)toxicological effects) and (iv) and the ‘potential implications’ of accepting the hypothesis (Additional file 1: Figure SI1 shows the GRACIOUS template).

By this approach the GRACIOUS framework generated several grouping hypotheses (both human and environmental) and tailored Integrated Approaches to Testing and Assessment (IATA) that support the user to gather the information needed to test the grouping hypothesis. The IATAs are structured as decision trees that incorporate decision nodes (DNs) (relevant questions) and guide the user to identify the information to be gathered or generated. To this end, a tiered testing strategy (TTS) that supports each DN has been proposed for different routes of exposure [5–8]. This TTS guides the user to identify relevant methods which increase in complexity with each tier level, starting from acellular/in vitro tests (Tier 1) to advanced cellular models (Tier 2) or in vivo assays (Tier 3). A data matrix is then generated according to the IATA to allow comparison of data across the group candidates, and grouping is confirmed or refuted through the assessment of similarity of NFs for each DN.

The detail and acceptability of the grouping varies according to the purpose (for regulatory implications low levels of data variability are accepted compared to precautionary/safe (r) by design, SbD, measurements). Once a provisional group has been generated, the user can fill data gaps (for the so-called target NFs) by the read-across of data from source NFs (or possibly non-NFs) for which the data supporting risk assessment is available.

To support grouping and read-across, a similarity assessment of the provisional group members is required. In the case of SbD/precautionary based grouping, a qualitative similarity assessment may be sufficient, based on expert judgment of the evidence available. Conversely, for regulatory purpose, a quantitative similarity assessment may be needed [9].

### Oral hypotheses and IATAs

By addressing the questions posed by the template, information on dissolution and hazard of orally ingested NFs were critically analyzed. Thus, the GRACIOUS project generated 9 different hypotheses to support grouping of NFs relevant to the oral route of exposure (H–O–I; H–O–Q1, H–O–Q3; H–O–S1, H–O–S2; H–O–S3; H–O–G1, H–O–G2; H–O–G3)[4, 6]. The complete list of human oral hypotheses (H–O–) with the related wording as well as the general template layout is reported in Additional file 1: Figure SI1.

**Fig. 1:**
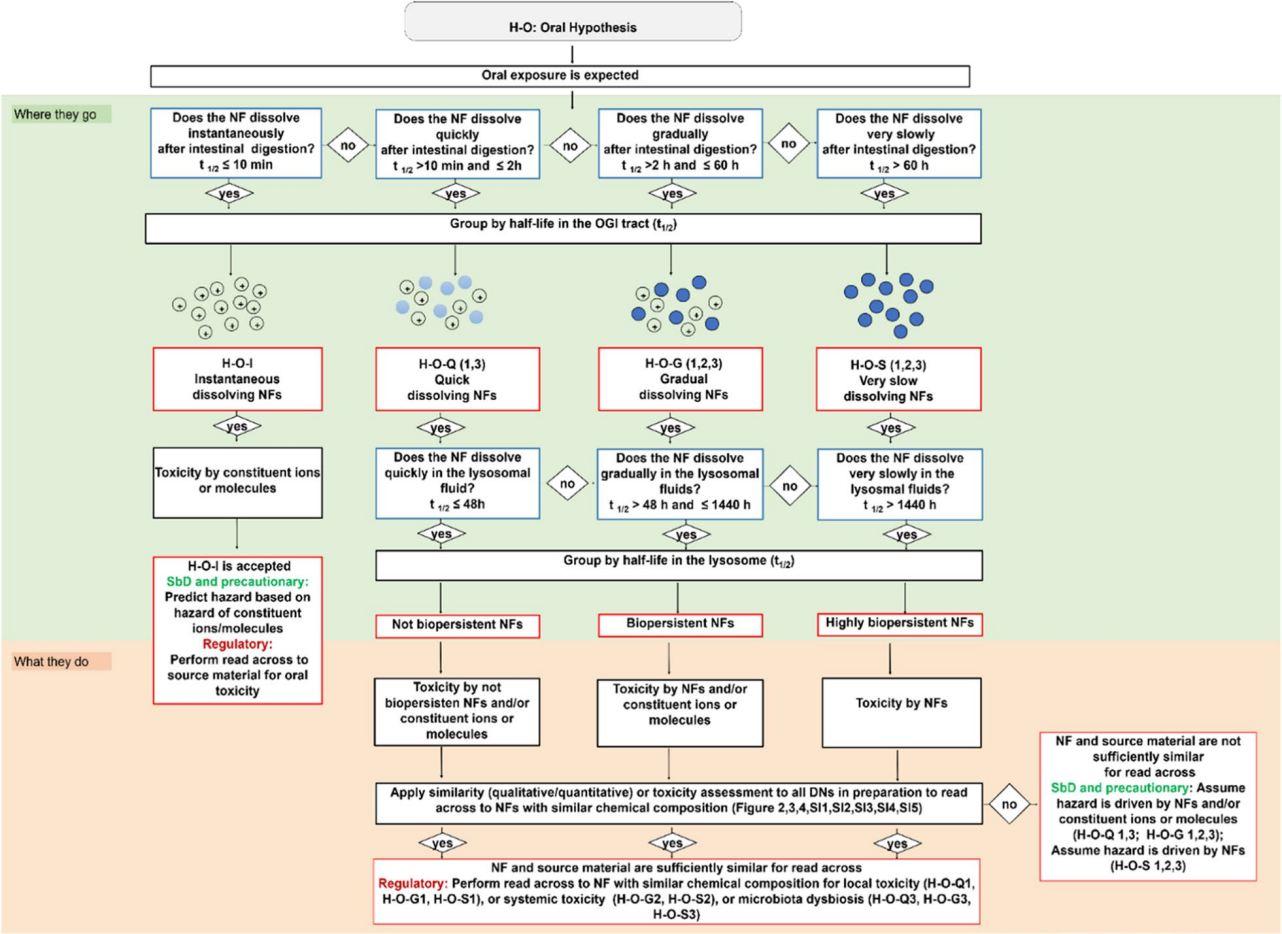
The oral IATAs generated by the GRACIOUS project [6] ( © MDPI, 2021) to support the grouping and read-across of NFs according to the GRACIOUS Framework [3]

According to the GRACIOUS framework, each of these hypotheses can be verified and the relative groups confirmed or rejected by the application of specific oral IATAs. Figure 1 reports the overall description of oral IATAs and summarizes all possibly hypotheses to which the testing refers (listed in the Figure). Moreover, it is possible finding the single oral IATA with the relative dissolution and hazard DNs for each of the oral hypotheses in Di Cristo and co-workers [6].

In detail, Fig. 1 shows the dissolution DNs that allow for measuring dissolution in the oro-gastrointestinal (OGI) and lysosomal simulant fluids thus supporting grouping of NFs based on the similarity of dissolution rates. Pragmatic cut-offs expressed as half-time values, spanning from 10 min to 60 h for the OGI fluids or from 48 to 1440 h for the lysosomal fluids, have been proposed for describing the dissolution kinetics in both the OGI and lysosomal compartments [6]. In the gastrointestinal tract, the dissolution kinetics allow identification of the potential contribution of ions and/or molecules (depending on the NF chemistry) *vs*. particles, and it links this to the likelihood of biopersistence of related nano-specific properties (*i.e.*, the retention of the nanoscale particle size) [6]. Accordingly, the hypotheses allowed for the generation of provisional groups that include NFs with similar dissolution rate in the OGI, namely instantaneous (H–O-I), quick (H–O-Q 1,3), gradual (H–O–G 1,2,3) and very slow (H–O–S 1,2,3) dissolving NFs (red boxes, Fig. 1). In the lysosome, the dissolution kinetics predict the potential of NFs to accumulate in secondary organs and to identify if the NFs may exert systemic toxicity due to accumulation and/or to the release of toxic ions or molecules. By the term ‘systemic toxicity’ we refer to toxicity to cells, tissues and organs that are exposed following translocation from the gastrointestinal tract. Accordingly, the hypotheses allowed for the identification of provisional groups that include NFs with similar potential to dissolve in the lysosomes, namely groups of not biopersistent, biopersistent or highly biopersistent NFs (Fig. 1, red boxes).

Lastly, the IATAs further investigated the toxicity possibly linked to the NFs by hazard descriptors (the hazard DNs) including cytotoxicity, barrier integrity, inflammation and/or genotoxicity (not reported in Fig. 1) by means of similarity assessment; the derived hazard driven groups, which are reported in Additional file 1: Table SI1, can then relate to the local toxicity exerted on the intestine barrier or on the microbiota and to the systemic toxicity to secondary targets organs (e.g., liver and kidneys) accordingly to the hypotheses (H–O–Q1,3; H–O–G1,2,3; H–O–S1,2,3) (Fig. 1, Additional file 1: Figure SI1, Table SI1).

Depending on the hypothesis selected, the oral IATAs allow for the testing of both toxicokinetic and hazard DNs by means of a TTS. Table 1 summarizes the biological endpoints which are requested by the TTS to address the questions posed by the DNs, dissolution and hazard descriptors (Table 1, green row), along with a list of assays/methods which growths in complexity as the Tier level increases (Table 1, blue rows).

**Table 1:**
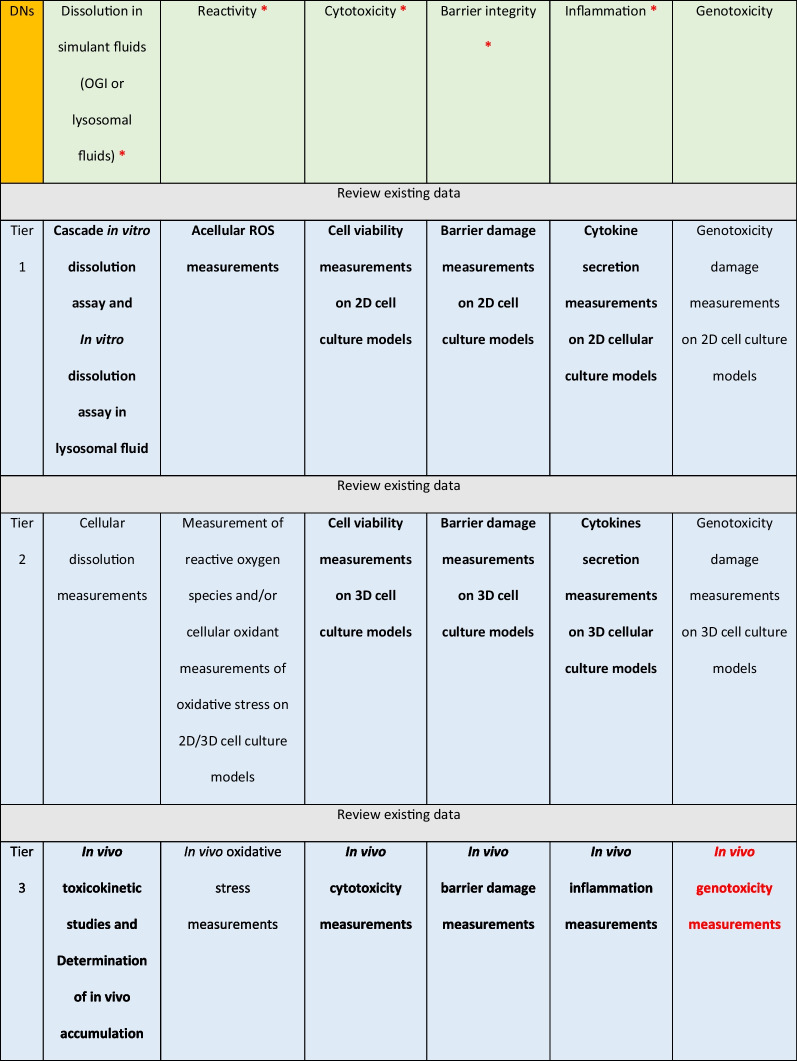
The DNs (green) (with relative biological endpoints) of the oral Integrated Approach to Testing and Assessment (IATA), and the tiered testing strategy (TTS) (blue) to support collection of evidence for grouping (Table adapted from [6] (© MDPI, 2021)

The red stars indicate the DNs addressed in this study. The bold text at Tier 1 and Tier 2 level indicates the assays performed to complete the data matrix required for the similarity assessment. The bold text at Tier 3 indicates the questions addressed by literature-based evidence

The case study presented in this paper specifically addresses the H–O–G hypothesis which accounts for grouping of gradually dissolving NFs and apply silicon dioxide, SiO_2_ NFs as a case study. This hypothesis can group NFs on the basis of the dissolution rate (both in the OGI and in the lysosome) which align with values for gradually dissolving NFs. It allows for the acceptance that NFs may exist as both NFs and constituent ions or molecules that may exert a certain toxicity. Two specific hazard driven hypotheses H–O-G1 and H–O-G2 are then selected herein and assessed through the corresponding oral IATAs. Some relevant information on the existing knowledge used to generate the H–O-G hypotheses is reported below.

### Silica NFs as a case study for gradual dissolving hypotheses (H–O–G): existing information

*Context and life cycle exposure:* Large-scale industrial production and commercialization of amorphous silica (SiO_2_) NFs have increased the risk of human exposure posed by these particles [10–12]. Humans can be exposed to silica NFs by different routes including ingestion from personal care products (e.g., lipstick and toothpaste), medicines and food. Most of the silica NFs can be found as food additives (E551) like anticaking agents, flavour enhancers, food pigments and health supplements [13]. Thus, the potential toxicity of such particles is of great interest. Importantly, only the amorphous form of silica is authorized as a food additive (E551). The amorphous forms permitted as food additive E551 include fumed (pyrogenic) silica and hydrated silica (precipitated silica, silica gel and hydrous silica) but not colloidal silica [14]. The food additive E551 is a material composed of aggregated nanosized primary particles that could further agglomerate into larger structures (greater that 100 nm). However, it cannot be excluded that some aggregates are smaller than 100 nm in size [14]. Moreover, there is still a chance that these agglomerates/aggregates could disagglomerate/disaggregate in body fluids into smaller, nanosized constituent particles. Although colloidal silica NFs are not intended for the purpose of oral consumption, we have included them in this study to investigate the suitability of the oral hypotheses, IATAs and TTS. As mentioned above, silica NFs can also be ingested as part of medicinal products. In this regard, mesoporous silicas are used as a carrier to improve oral bioavailability or to allow for more controlled drug release [15, 16]. For this reason, also the mesoporous silicas were utilized as case study materials to test the applicability of the oral IATAs.

*What they are; Where they go; What they do:* the collected data informed us that the emergent hazard linked to SiO_2_ NFs were associated to local toxicity (effects exerted on the intestinal barrier) and systemic toxicity (effects exerted on secondary target organs owing to accumulation) [6]. Emerging evidence also highlighted a hierarchy of biodurability and persistence for metal oxide NFs, which place the SiO_2_ NFs between titanium NFs and zinc NFs (titanium NFs > silica NFs > zinc NFs [17]) identifying the silica based NFs as gradually dissolving NFs. For the explained reasons, we selected the case study of SiO_2_ NFs in order to verify the H–O–G1 and H–O–G2 hypotheses.

The toxicokinetic and hazard DNs which are relevant for the case study are marked as red star and in bold in Table 1. Here, at Tier 1 and Tier 2 level de novo data has been produced, whereas at Tier 3 level the results presented are literature-based. Therefore, the application of the IATA and the associated TTS resulted in generation of a data matrix that included in vivo pre-existing data for all the selected SiO2 NFs and DNs, leading to a gap analysis. Completion of the data matrix using available data revealed an existence of some in vitro and in vivo data, allowing a gap analysis to be conducted. In vitro studies were conducted to fill the gaps to ensure a full complement of in vitro data to assess similarity prior to application of read-across of the in vivo data (if the grouping hypothesis is accepted).

To summarize, the aims of the study were:

(i) To use a panel of silica NFs, differing in size, composition, surface coating and synthesis technique, to assess whether the existing formulated hypotheses (H–O–G1 and H–O–G2) could be verified through the corresponding IATAs and used to support the grouping process.
(ii) To assess the suitability of the proposed Tier 1 and/or 2 methods to generate the dissolution and hazard information required by the IATA DNs to support decision making. This suitability assessment includes analysis of the data reliability to ensure further robust assessment of similarity (see iii).
(iii) To apply similarity algorithms to support a quantitative similarity assessment among the tested DNs (marked with a red star in Table 1) and the corresponding Tier 1 and/or Tier 2 selected assays (marked as bold in the Table 1) of the oral IATA.

In this paper the strengths and weaknesses of the oral IATAs and their flexibility are outlined and how they can be utilized to support grouping and read-across decision making.

## Results

### Basic information on physicochemical features of NFs

The basic PC properties of the silica NFs selected for this case study are reported in Table 2 together with newly generated data. The panel included five amorphous silica NFs (NM-200, NM-203, Silica-Std, Silica-Al, Silica-Silane) of comparable constituent particles with size of ca. 10–14 nm and specific surface area (175–216 m^2^/g) (as measured in water after the synthesis and reported by the manufacturers) [18, 19]. These silica NFs are produced following different methods, which are based on wet chemistry (precipitation or ion exchange/colloidal) or on thermal processes (pyrogenesis). Different surface treatments (non-surface modified, silane or aluminate treated) are also reported for all the NFs. The two mesoporous MCM-60 and MCM-170 presented constituent particles with size of about 60 and 170 nm, respectively and a specific surface area from 1110 to 1650 m^2^/g [20].

**Table 2:** The panel of silica NFs used for the case study to assess the performance of the GRACIOUS oral IATA to support grouping of NFs

	Crystallinity	Synthesis method	Surface Treatments	Shape	Specific surface area (BET, m2/g)	Constituent particle size (nm, average ± SD) by Manufacturers	Constituent particle size (nm, average ± SD) experimentally measured
NM-200	Amorphous	Wet (precipitation)	None	Spheroidal	189	14 ± 7	20.7 ± 6.2
NM-203	Amorphous	Thermal (pyrogenesis)	None	Spheroidal	203	13 ± 6	13.3 ± 4.1
Silica-Std	Amorphous	Wet (colloidal)	None	Spheroidal	209	10	10.0 ± 2.1
Silica-Silane	Amorphous	Wet (colloidal)	Surface modified with glycerol-propyl moieties from alkyl-tri-alkoxysilanes	Spheroidal	216	10	12.1 ± 2.4
Silica-Al	Amorphous	Wet (colloidal)	Surface modification with Na-aluminate	Spheroidal	175	11	13.3 ± 4.1
MCM-60	Mesoporous	Wet (colloidal)	None	Spheroidal	1647	60 ± 10	69.5 ± 12.8
MCM-170	Mesoporous	Wet (colloidal)	None	Spheroidal	1111	165 ± 21	151.2 ± 71.0

The information provided includes both basic PC characteristics and synthesis routes of the different silica NFs as described by the manufacturers. The last column refers to the TEM size analysis of constituent particles in water as experimentally provided in the work. The obtained data are compared to the manufacturers’ values

In this study, Transmission Electron Microscopy (TEM) analysis in water of constituent particles confirmed that the size and the morphology of the NFs corresponded to those declared by the manufacturers (Table 2 and Additional file 1: Figure SI2, left column). TEM images indicate that most of the particles (e.g., Silica-Silane, Silica-Std and Silica Al) are still recognizable as individual particles, while the other NFs appear interconnected to each other by supramolecular structures which likely indicate the formation of agglomerates that include the constituent particles (in line with Dynamic Light Scattering, DLS data, see next). When NFs are suspended in the cell culture medium (Minimum Essential Medium, MEM, supplemented with 2 mM L-glutamine is used here as representative cell culture medium), all the NFs (except MCM-170) appear organized as agglomerates including constituent particles (Additional file 1: Figure SI2, right column). Furthermore, the inspection analysis, although qualitatively, supported no evident biotransformation of the silica NFs in the medium (i.e., dissolution) as they are still visible in a quasi-spherical form upon incubation of size comparable to the pristine size. For MCM-170 a limited colloidal stability in suspension was observed with the suspended particles presenting, in some cases, a size reduction, possibly indicative of de-agglomeration and/or dissolution.

Hydrodynamic size distribution analysis and dispersion stability of NFs were performed by DLS over a period of 24 h, according to the timing of the employed in vitro experiments. Additional file 1: Figure SI3 reports the DLS spectra obtained at t_0_ and t_24_ for all the NFs in cell culture medium. The results indicate that in MEM, at t0, the medium size range, D_H_, of NFs roughly corresponds to those described in water. In contrast, at 24 h, there is a tendency for larger agglomerates, ranging from 100 to 2000 nm, with PDI values gradually approaching 1 (not shown). In some cases (MCM-170 and NM-203), smaller particles are observed with a corresponding higher background noise (Additional file 1: Figure SI3 and Table SI2). These data combined suggest that the agglomeration structures are stable initially and well dispersed in the suspension until they gradually settle down or de-agglomerate. It is reported that the background noise can increase for diluted suspensions [21]. Here, we observe an increase of the background noise, but we performed repeated DLS measurements on a suspension at constant concentration. This allowed us to attribute this effect (although only based on qualitatively observations) to an actual dilution of a suspension due to particle deposition. In any case, at 24 h corresponding to the last point of the cellular in vitro experiments, all NFs appear organized in stable agglomerates that are still detectable in solution by DLS (Additional file 1: Figure SI3 and Table SI2), although, NM-203 and MCM-170 demonstrate a different pattern visible by DLS at comparable experimental conditions in the cell culture medium (Additional file 1: Figure SI3 and Table SI2).

If we consider the constituent particles and their relative sizes declared by the manufacturers, a 15-fold primary size difference is noted between the smallest (Silica-Std and Silica-Silane) and the largest (MCM-170); whereas a ninefold difference is observed between the lowest surface area (Silica-Al) and the highest (MCM-60). In cell culture medium, pristine particles are organized in stable agglomerates hence the average surface area will be possibly larger.

In conclusion, the NFs selected for the case study differ in size, surface areas, sedimentation, surface coating, and synthesis methods (Table 2). Such differences may have an impact on the answers to question posed in the DNs of the oral IATAs [21]. Therefore, in the light of quantifying similarities among NFs, (one of the aims of this work), the similarity assessment should include different PC properties that characterize the selected panel of silica NFs.

### DN addressing dissolution of NFs in OGI fluids: data generation and interpretation

**Fig. 2:**
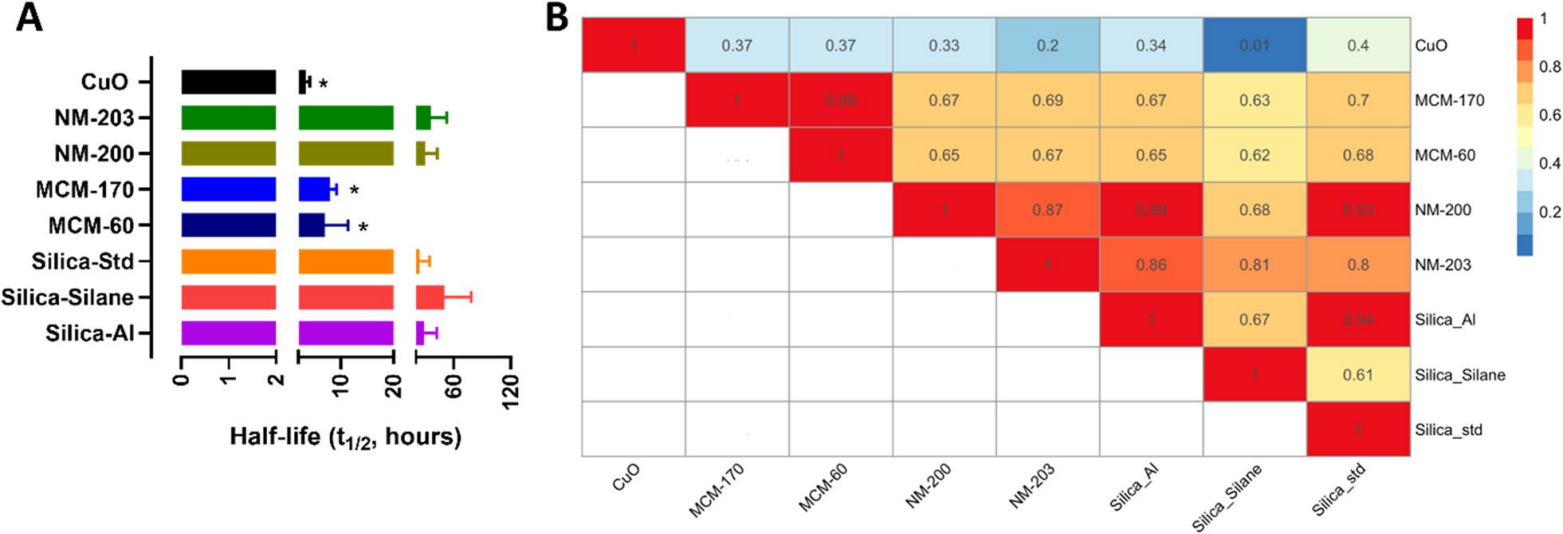
**A** Dissolution half-time (t_1/2_) of the selected NFs (1 mg/mL) measured after 155 min of OGI digestion (= after 30 min of intestinal phase). Data are expressed in hours and as mean ± standard deviation (n = 3) * p ≤ 0.05 vs Silica-Silane. **B** Similarity assessment by Bayes Factor (BF) pairwise analysis for selected NFs (value are scaled between 0 and 1) based on % of NF dissolution (ratio between % ‘dissolved’ of one NF by the other) together with the half-time values in OGI fluids. Values close to zero (blue color) indicate that the NFs are not similar and values close to one (red color) indicate similarity between NFs

The oral IATAs start with a common DN which measures the dissolution kinetics of NFs in OGI fluids and establishes groups of NFs which show similarities in the dissolution kinetics (Fig. 1) [6]. Hence, following the TTS of this DN, we measured the dissolution kinetics by applying an in vitro cascade dissolution assay that includes the consecutive addition of simulant OGI fluids (saliva, stomach and intestine) to the NFs [22–24]. According to EFSA guidance [25] the measurement of dissolution rate is required after at least 30 min from the addition of the intestinal simulant juice. Data is expressed as half-time (t_1/2_) values (reported in Fig. 2A) and as % of dissolution (Additional file 1: Table SI3).

According to the dissolution cut offs of the oral IATA (Fig. 1), the obtained values confirm the provisional assignment of the silica NFs in the group of ‘gradually dissolving’ (H–O–G), having quantified t_1/2_ values which span from 7 h (MCM-60) to 50 h (Silica-Silane) (Fig. 2A). Interestingly, also the copper (CuO) NM, used as a positive control to test the hazard DNs (see next), belongs to the same group of gradual dissolving NFs showing a t_1/2_ of around 3 h.

Therefore, the GRACIOUS grouping hypotheses relevant to silica NFs would be:

- NFs with a gradual dissolution (H–O–G1): Following oral exposure, both NFs and constituent ions or molecules may lead to local inflammation in the OGI tract (Additional file 1: Figure SI1).
- NFs with a gradual dissolution (H–O–G2): Following oral exposure, both NFs and constituent ions or molecules may translocate to secondary target organs and will lead to systemic toxicity in secondary organs (Additional file 1: Figure SI1).

### DN addressing dissolution of NFs in OGI fluids: similarity assessment

Statistical analysis via ANOVA of the dissolution half-time values identified significant differences between the slower dissolving NF (Silica-Silane), the faster dissolving silica NFs (the MCMs) and the CuO NM (Fig. 2A).

To better quantify the lack of similarity in dissolution values, we applied the BF pairwise approach which compares pairs of NFs by estimating the similarity of dose–response curves, here formed by the dissolution % and the half-time curves (Fig. 2B). The values presented are scaled between 0 and 1, where 1 indicates identical curves, while values above or equal to 0.7 indicate highly similar NFs [26]. The yellow and light orange colors (from light orange: 0.7 to yellow: 0.5) represent the comparison of mesoporous NFs to the other NFs suggesting that these silica NFs cannot be considered as a single group *i.e.,* they are not highly similar according to the BF analysis but as two groups. In addition, Silica-Silane demonstrates low similarity (from light orange to yellow) to NM-200, Silica-Std and Silica-Al, whereas it demonstrated a high level of similarity to NM-203. Nevertheless, the other amorphous silica NFs are very similar to each other (deep orange and red colors, BF values ≥ 0.7). On the other hand, CuO NM is not similar to the silica NFs, with a range of colors spanning from green to deep blue (BF values < 0.5), highlighting differences between materials of different chemistry.

Interestingly, the BF pairwise analysis (Fig. 2B) is in agreement with the cluster analysis (Additional file 1: Figure SI4), where the data partitions into two sub-groups of silica NFs are clearly evidenced in the x-axis which indicate again a group corresponding to the MCM NFs (MCM-170 and MCM-60) and the other to the amorphous silica NFs. Therefore, despite the pragmatic cut-offs established for the dissolution data [6] which suggests all of the SiO_2_ NFs studied fall within the ‘gradually dissolving’ group, the similarity algorithms applied suggests that the MCM silica NFs could form a separate group as they are not sufficiently similar (with respect to dissolution rate data in OGI fluids) to the amorphous NFs, according to the thresholds set by the BF pairwise analysis (BF values < 0.7).

### DN for assessing dissolution in PSF: data generation and interpretation

According to H–O-G2 of the oral IATAs [6] (Fig. 1 and Additional file 1: Figure S1), when silica NFs show similarities in PSF dissolution kinetics, they can be grouped according to their potential to accumulate in cells. Following the TTS associated with this DN, the lysosomal dissolution was assessed by means of a dynamic dissolution test (ISO/TR 19,057:2017) which employs a simulant lysosomal fluid at pH 4.5. According to H–O-G2, similar NFs can form a group if they exhibit a *t*_*1/2*_ > 48 h and a t_1/2_ < 1440 h (Fig. 1) [6, 7].

**Fig. 3:**
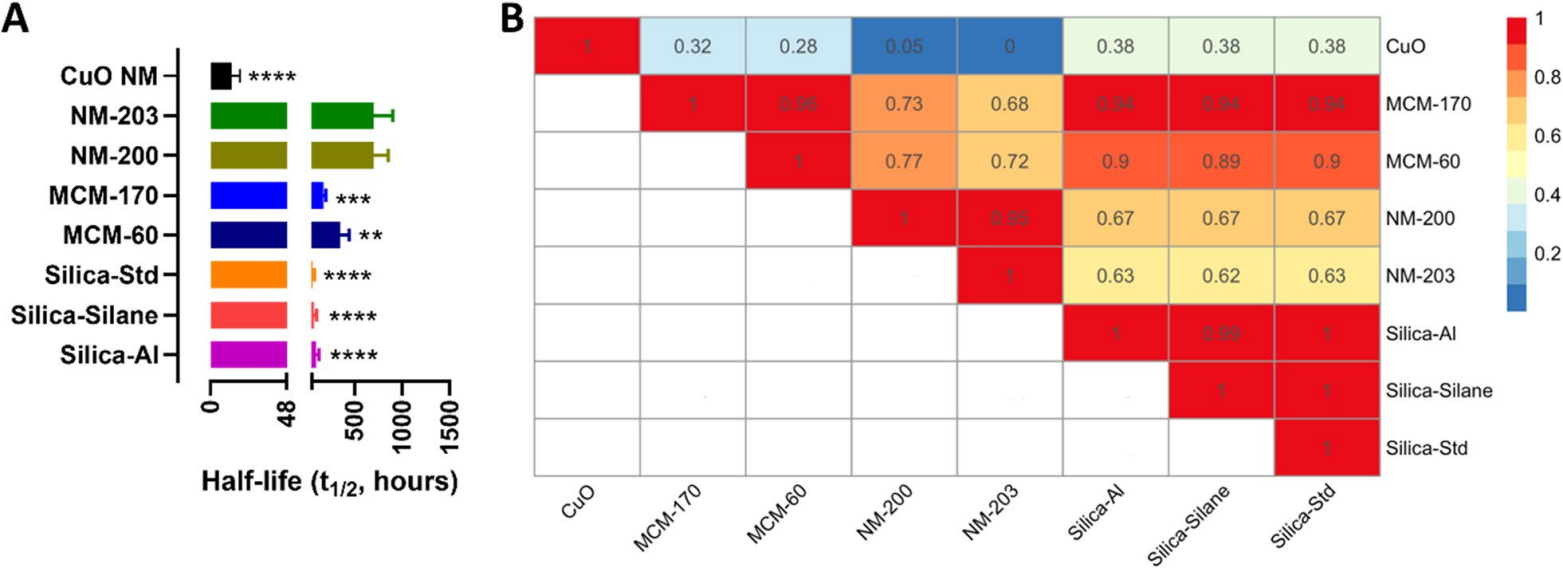
**A** Dissolution half-time (t_1/2_) of the selected silica panel (1 mg) measured in PSF fluids. Data are expressed in hours and as mean ± standard deviation (n = 3). **p < 0.01, ***p < 0.001 and ****p < 0.0001 versus both NM-200 and NM-203 **B** Similarity assessment by BF pairwise analysis for selected NFs (value are scaled between 0 and 1) based on NF dissolution rate (ratio between rate ‘dissolved’ of one NF by the other) together with the half-time values in PSF fluids. BF Values close to zero (blue color) indicate that the NFs are not similar and BF values close to one (red color) indicate similarity between NFs

Figure 3A shows the measured t_1/2_ values for all silica NFs (data expressed as dissolution k rate are reported in Additional file 1: Table SI4) which falls between 48 and 1440 h, in line with ‘gradually dissolving’ in the PSF fluid and possibly ‘accumulating and biopersistent particles’. Interestingly, the CuO NM does not belong to the gradual dissolving group as it has a half-time value of around 13 h, thus it appears to be a quick dissolving material in the PSF fluids (and thus not biopersistent) and as consequence is unlikely to accumulate as a particle accordingly to the definition of quick dissolving NF in lysosomal fluids (Fig. 1) [6, 7].

### DN for assessing dissolution in PSF: similarity assessment

Statistical analysis via ANOVA of the dissolution half-time values identified significant differences among the NM-200/NM-203 NFs, the remaining silica NFs (Silica-Std, Silica-Silane, Silica Al and MCM-60, MCM-170) and the CuO NM (Fig. 3A).

To further explore these differences, we applied the BF pairwise approach (Fig. 3B). Interestingly, the BF approach confirmed a lack of similarity across all silica NFs showing two sub-groups: the slower dissolving and more accumulating NFs (NM-200/NM-203) versus the more rapidly dissolving and less accumulating NFs including the Silica-Std, Silica-Al and Silica-Silane and the MCMs NFs (all red/orange *vs* the others which are in light orange and yellow scale indicating dissimilarity). The CuO NM appears highly dissimilar from all the tested silica NFs (from green to deep blue BF values).

The BF pairwise analysis (Fig. 3B) is in agreement with the cluster analysis which further reported differences between NM-200 and NM-203 and the other tested NFs (Additional file 1: Figure SI5). Here, the similarity tool indicates on the x-axis, 3 sub-groups of silica NFs identified as: NM-200 and NM-203; Silica-Std, Silica-Al and Silica-Silane; and the mesoporous silicas MCM-60 and MCM-170. An alternative group is also visible, and it has been associated to the CuO NM that seem to be closer to the colloidal silica groups compared to the other silica NFs as also evidenced by the BF approach. Therefore, despite all silica NFs falling within the parameters defining ‘gradually dissolving’ in PSF fluid, as postulated by the cut-offs defined within the H–O-G2, the similarity algorithms applied on experimental data showed that the whole panel of silica NFs could be further subdivided into multiple groups, according to the BF pairwise analysis (BF values < 0.7).

### DN addressing local tissue hazard of NFs (H–O–G1): data generation and interpretation

We have assessed the local toxicity using undifferentiated intestinal Caco-2 cells (Tier 1) and a 3D intestinal triple co-culture of Caco-2, mucous-secreting HT29-MTX and lymphoblast-like cells Raji B cells (Tier 2). We selected these models according to the indications as provided by the TTS [6] and because they are widely employed models to mimic the functionalities of the intestinal epithelium [27, 28]. Undifferentiated Caco-2 cells were treated with the selected silica NFs for 24 h with a broad range of concentrations (from ca.1 µg/mL to 100 µg/mL). These values are widely applied for toxicology assessment of NMs [24, 29, 30]. Moreover, this range covers a realistic and worst-case scenario of daily human consumption of silica NFs (see supplementary file for further information). We tested only silica NFs (and not the ionic fractions) as we do not expect toxicity by the ionic fractions. Indeed, the release of silicic acid it is generally recognized inherently biocompatible [31] and should not be the main driver of any toxicity; hence, the dissolution descriptor (in the OGI and lysosome) will be related to the hazard outcomes only considering its potential predictivity to represent the likelihood of NF durability (in terms of retention of nanoscale size) as the release of silicates ions via dissolution cannot be relevant to lead to toxicity in this specific case study. The reported concentrations refer to the nominal dose *e.g.,* the mass of NFs per volume of suspension. Nominal concentrations were selected instead of the effective dose (*e.g*., actual mass of NFs that affect the cells) because recent findings demonstrate that for low density, soluble agglomerates of silica NFs, the sedimentation occurs generally in larger timeframes (*e.g.* more than 140 h) [21] than those applied for the cells (i.e., 24 h). Thus, in such temporal window the particles should mostly be suspended in the biological medium. In this case, recently, it has also been shown that beyond sedimentation, these NFs indeed usually develop convention forces which allow for the majority of the particles to reach randomly the target cells thus exerting their potential toxicity as normally occurs for soluble drugs (which exert their toxicity in any case without sedimenting) [32, 33]. Our data indicate (both by the DLS and TEM size analyses) (Additional file 1: Figure SI2, SI3) the presence of relative stable silica NFs in the form of pristine or agglomerates particles (with a small deviation only for NM-203 and MCM170) within the temporal framework applied for the cellular experiments (24 h). This evidence alongside the above considerations leads us to apply the nominal dose instead of the effective dose.

The assay selection to test the H–O-G1 hypothesis is made accordingly to the TTS of the oral IATA [6], where the Alamar Blue (cytotoxicity), the transepithelial/endothelial electrical resistance (TEER) measurements (barrier integrity) and the release of IL-8 cytokine secretion (pro-inflammatory signalling) are some of the suggested methods. For instance, the Alamar blue assay is a validated method to screen cell viability [29]; TEER assessment is considered a good marker for the monitoring of the integrity of the intestinal tissue and can be used to replace in vivo histopathologic analysis [6] and lastly, IL-8 cytokine release was instead found one of the most sensitive markers for assessing pro-inflammatory responses on cell models, including also Caco-2 cell based intestinal in vitro models such as monocultures and co-cultures [27, 28, 34]. The same assays and doses (except for TEER measurement) were also applied to test the H–O-G2 hypothesis for the above explained reasons (see next).

**Fig. 4:**
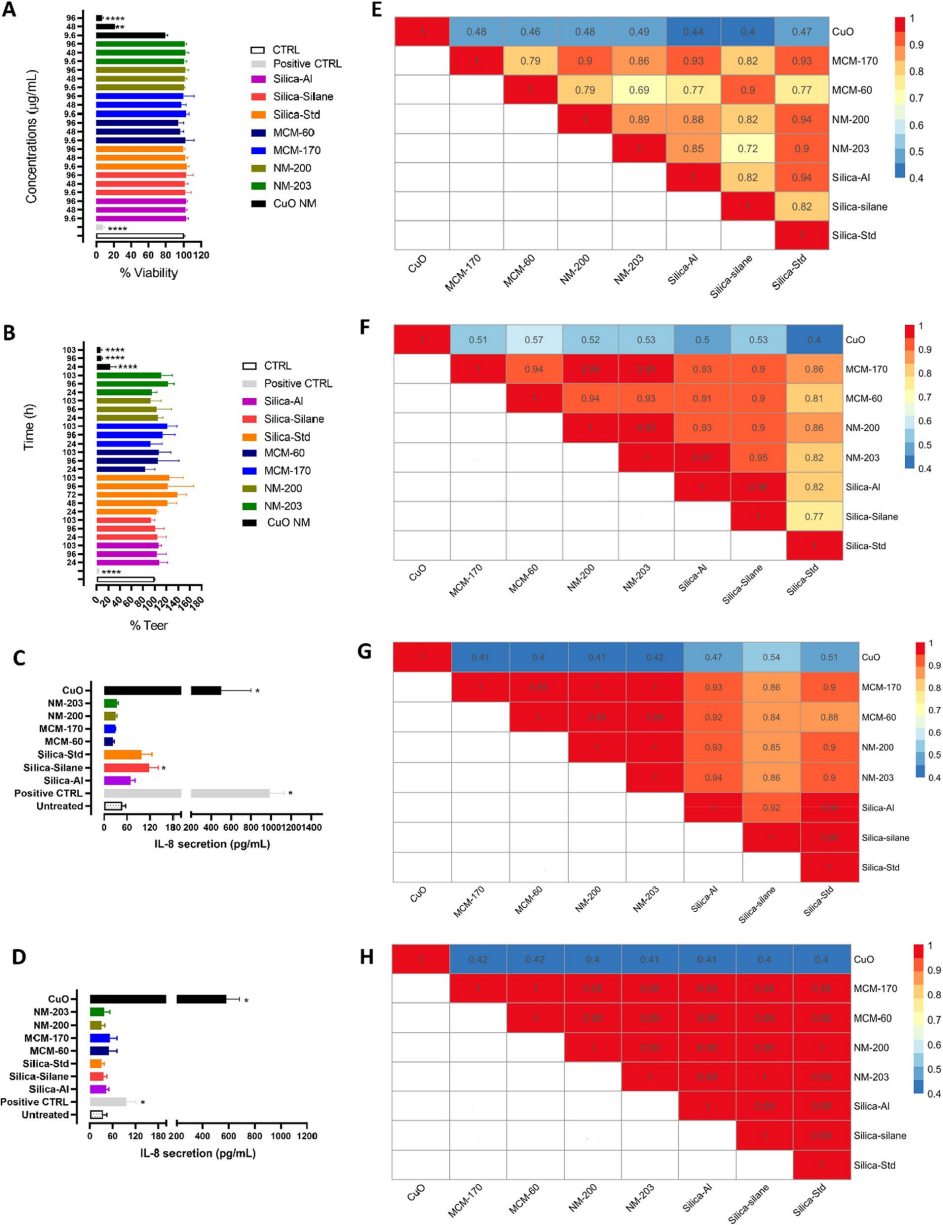
Impact of silica NFs on intestinal models. **A** Undifferentiated Caco-2 cells were cultured in a 96 well plates for 24 h and exposed to various concentrations of silica and CuO NM (from 0.98 to 125 µg/mL) for 24 h followed by the assessment of viability using Alamar blue assay. **B** Impact on barrier integrity of triple culture model measured daily for 5 days during exposure of NFs (33.6 µg/mL) using TEER (only selected days are shown). **C** Undifferentiated Caco-2 IL-8 production after 24 h of exposure to 48 µg/mL of NFs and **D** IL-8 production assessed on the fifth day apical supernatant of 3D model using Enzyme-linked immunosorbent assays (ELISA) exposed to 33.6 µg/mL of NFs. **E**, **F**, **G** and **H** BF pairwise similarity assessment was conducted comparing the curves of % viability, % of TEER value, undifferentiated Caco-2 cells and triple culture IL-8 production measured in pg/mL respectively, together with the concentration values and different time points (TEER measurements). Values range between 0 and 1 (similar NFs). Values close to zero indicate that the NFs are not similar. Data are shown as mean ± standard deviation. * p ≤ 0.05 vs untreated. Triton X100 (0.01%) was used as positive control for viability and TEER experiments, whereas TNF-α (2 µg/mL) was used as positive control for cytokine secretion

Results are reported in Fig. 4A (only three representative concentrations, i.e., 9.6, 48 and 96 µg/mL) and in Additional file 1: Figure SI6 (all the concentrations are shown). The silica NFs did not induce significant toxicity to the monoculture Tier 1 model according to the Alamar Blue assay at any tested concentration and moreover no significant differences were identified among the tested silica NFs. In comparison, the positive control CuO induced a dose dependent decrease in cell viability (Fig. 4A and Additional file 1: Figure SI6).

As the silica NFs were not toxic to the undifferentiated Caco-2 cells, the 3D model was treated with three selected concentrations (6.72, 33.6 and 67.2 µg/mL) of particles, daily for 5 days to cover a worst-case scenario of daily human consumption of silica NFs (for more details refer to supplementary information). TEER measurements (only for the 3D models) and IL-8 secretion (for both models) are reported only for selected concentrations (Fig. 4B–D). Data was also acquired for the other exposure concentrations and times (for TEER measurements) and was comparable to that reported in Fig. 4 (Additional file 1: Figure SI7).

In the experimental conditions applied, none of the silica NFs had any significant deleterious effect on barrier integrity (Fig. 4B and Additional file 1: Figure SI7), while the positive controls (CuO NM and Triton X100) both decreased TEER within 24 h of exposure and remained low for the duration of the 5-day study. Similarly, IL-8 production by the Tier 1 monoculture of Caco2 cells was not increased above control values for all of the NFs with the exception of Silica-Silane, although the IL-8 production generated was very small and possibly below biological significance (Fig. 4C). IL-8 production for the Tier 2 method, using the 3D cultures was also assessed. Again, none of the NFs, including the Silica-Silane, were able to induce any significant changes in IL-8 expression by the 3D culture (Fig. 4D). In contrast, the positive controls, CuO NM and TNF-α both induced a significant increase in IL-8 production in both the Tier 1 and Tier 2 culture models (Fig. 4C–D).

The different toxicity of the silica and CuO NMs were also confirmed by the testing of surface reactivity (Additional file 1: Figure SI8). Indeed, we measured a high level of reactivity only for the CuO NM. These data further confirm the low toxicity of silica NFs in comparison to CuO NMs whose toxicity is well established [35].

### DN addressing local tissue hazard of NFs (H–O–G1): similarity assessment

When applying the pairwise similarity assessment by BF calculation to the data for all the studied biological endpoints, all silica NFs appear similar (from red to orange) (Fig. 4E–H). However, a few yellow boxes are visible (BF ≤ 0.7), and they represent the comparison of MCM-60 and Silica-Silane to NM-203. Such small differences can be considered negligible as they are not detectable when assessing at the Tier 2 level.

The same happens when comparing the IL-8 response in the Tier 1 (Fig. 4G) and Tier 2 (Fig. 4H) models, in which the Tier 2 model increases the confidence of the level of similarity of the silica NFs to each other in terms of their lack of ability to induce production of the pro-inflammatory mediator. Note that the silica NFs panel appear dissimilar to the CuO NM across all the DNs addressed (dissolution and hazard) (Fig. 2 and 4), although within the same dissolution group (Fig. 2A). Hence, the Tier 1 and 2 hazard data indicate that all the silica NFs belong to the same group, demonstrating no significant local toxicity or pro-inflammatory response.

### Existing Tier 3 in vivo data required to test the H–O–G1 hypothesis

In vivo (Tier 3 level) data from the literature were analyzed to fulfil the TTS and to provide scientific based evidence to calibrate the Tier1 and Tier2 results of the H–O–G1.

The obtained information together with the presented results are interpreted to support the oral grouping decision. Table 3 summarizes all the in vivo studies collected. Only two publications where rats were orally exposed to NM-203 investigate local toxicity for silica NFs [36, 37]. Tarantini et al. reported no histological findings in the duodenum and colon of rats treated for 3 days by oral gavage to NM-203 (doses of 5, 10, or 20 mg/kg bw) [37]. Tassinari et al. show a qualitative histological analysis of small intestine, reporting no statistically significant findings after a 90-repeated exposure of rats by oral gavage to different doses of NM-203 (2, 5, 10, 20 and 50 mg/kg/bw) [36]. The lack of pathology observed in these two studies aligns well with the low toxicity observed in the in vitro studies. Moreover, it suggests that the selected in vitro models seem to be quite predictive of the in vivo data. Although a negative cannot be proven, the detrimental effect of CuO NM is clearly evidenced by our data. Moreover, the predictivity of our in vitro models is also confirmed by an in vivo study where the local toxicity of the same CuO NMs was analyzed for 5 consecutive days by oral gavage of rats at doses from 1 to 32 mg/kg/bw [38]. Here, it is reported that the CuO NM induced morphological alterations in the stomach (associated to a submucosal glandular inflammation) and in the intestine, thus confirming the toxicity of CuO observed in the in vitro experiments.

**Table 3:** In vivo data available to substantiate the oral grouping hypotheses, H-O-G1 and H-O-G2

NF	Model	Dose	Experimental design	Particles/Ions accumulation **	Effects		Reference
					Local	Systemic	
SiO_2_ (NM-203)	Rats	5, 10, or 20 mg/bw*	3-repeated exposure by oral gavage (sub-acute)	Not assessed	No histological changes of duodenum and colon; weak genotoxic effects in the colon	No histological changes of tissue samples from spleen, liver and kidney	[37]
CuO (Plasma Chem, GmbH)	Rats	1, 2, 4, 8, 16, and 32 mg/kg bw	5-repeated exposure by oral gavage (sub-acute)	Copper ions detected in liver, lung, kidneys, spleen, thymus, and mesenteric lymph node tissue	Histological changes in stomach where inflammation was observed	Alterations in the level of alkaline phosphatase (ALP) and aspartate aminotransferase (AST) liver enzymes; Histological changes in liver and bone marrow; Liver exhibited slight Kupffer cell hypertrophy/ hyperplasia and inflammation; Bone marrow changes included slight increased myeloid elements and decreased erythroid elements	[38]
SiO_2_ (Levasil® 200; Levasil® 200 PEG treated; Levasil® 200 phosphate treated; Levasil® 200 amino treated)	Rats	1000 mg/kg/bw	28-repeated exposure by oral gavage (sub-acute)	Not assessed	No adverse effects for any of the tested silicas (histopathological examination)	No adverse effects for any of the tested silicas (clinical pathology, clinical chemistry, acute phase proteins and metabolome analysis)	[39]
SiO_2_ (mesoporous silicas of two target sizes, 100 and 300 nm)	Mice	100 and 1000 mg/kg/bw	5-repeated exposure by oral gavage (sub-acute)	Silica particles detected in jejunum	No histological changes of small intestine; No intestinal inflammation	No histological changes of liver	[40]
SiO_2_ (NM-203)	Rats	2, 5, 10, 20 and 50 mg/kg/bw*	90-repeated exposure by oral gavage (sub-chronic)	Silicon ions detected in liver and spleen	No histological changes of small intestine	No alteration in AST, Alanine aminotransferase (ALT), blood urea nitrogen (BUN) and creatinine serum levels; No histological changes of spleen, adrenals, Kidneys and thyroid; Different alterations were reported in the liver (hepatocyte vacuolization/steatosis, intralobular lymphoid infiltration, enlarged sinusoids and congestion of sinusoids); Histomorphometrical alterations in spleen; Different alteration in the immunotoxicity markers in the blood	[36]
SiO_2_ (NM-200)	Mice	4.8 mg/kg/bw*	18 months via drinking water (chronic)	Silicon ions detected in liver and kidney	Not assessed	Histomorphological alterations were identified in kidneys (vacuolization of tubular epithelial cells); Liver inflammation coupled to amyloidosis lesions	[41]
SiO_2_ (NM-203)	Rats	20 mg/kg/bw	1 and 5-repeated exposure by intravenous administration (acute and sub-acute)	Not assessed	Not assessed	Splenomegaly accompanied by inflammatory granulomas; Granulomas in liver parenchyma	[42]
SiO2 (mesoporous silicas of 75 nm)	Mice	50-100-200 mg/kg/bw	14-repeated exposure by oral gavage (sub-acute)	Silica particles detected outside the intestinal tissue and in the cytoplasm; Silicon ions detected in heart, liver, spleen, kidney, colon and intestine	Infiltration of inflammatory cells in the intestines; Intestinal oxidative stress and colonic epithelial cell apoptosis; no sign of genotoxicity in intestine	Increase of ALP, ALT and AST serum levels; Infiltration of inflammatory cells in the spleen	[43]
SiO_2_ (two colloidal silicas of 46 and 432 nm and mesoporous silicas of 466 nm)	Mice	100–300 mg/kg/bw (colloidal silicas); 100 mg/kg/bw (mesoporous silicas)	Single dose by intravenous administration (acute)	Not assessed	Not assessed	Tissue injury of heart, lungs, kidney, liver and spleen	[44]
SiO_2_ (NM-203)	Rats	2, 5, 10, 20 and 50 mg /kg/bw*	90-repeated exposure by oral gavage (sub-chronic)	Not assessed	Not assessed	Weak genotoxic effect in the spleen	[45]
SiO_2_ (NM-203)	Rats	2, 5, 10, 20 and 50 mg /kg/bw*	90-repeated exposure by oral gavage (sub-chronic)	Not assessed	Not assessed	No genotoxic effects in reproductive system (male and female)	[46]

Acute studies involve a single exposure with endpoints assessed at 24 h, the sub-acute studies involve repeated exposures for between 24 h and 28 days, the sub-chronic exposures include repeated exposures for 90 days, while the chronic studies involved repeated exposures for 6–12 months

*Silica dose relevant to daily intake [47]; **Ion accumulation refers to total ion content as measured in the relative organs by ICP-MS analyses

In vivo data on rats is also available for four different Levasil® 200 NFs [39], including the Levasil® 200 without any surface modification. This NF is similar in terms of production process to the colloidal Silica-Std used in our study. None of the tested Levasil® 200 NFs induce local toxicity after daily administration over a period of 4 weeks by oral gavage (1000 mg/kg/bw) [39]. In line with these data, a recent in vivo study on mice exposed to mesoporous silica NFs of two target sizes (100 and 300 nm) did not show any local toxicity (measured by histopathology and the evaluation of pro-inflammatory cytokines in the intestinal mucosa) after 5 consecutive days of oral gavage administration (100 to 1000 mg/kg/bw) [40]. Notably, one of the tested mesoporous silica NFs exhibited a similarly high specific surface area (SSA) (800 m^2^/g) and size (100 nm) to the mesoporous silica NF employed in the present study (MCM-170) (thus this article is considered highly valid for the application of a read-across). It is worth mentioning that a sub-acute study (14-repeated exposure) using different MCM silicas (75 nm with a very high SSA of around 40,000 m^2^/g) at doses of 50–100–200 mg/kg/bw showed intestinal oxidative stress and colonic epithelial cell apoptosis in treated mice [43]. The SSA of this MCM is 25-fold higher than that of MCM-60. Hence for the read-across applicability of this paper, further studies based on using MCM of the same size and increasing SSA will be required to establish a clear application domain. For this reason, this article was not considered for the read-across.

Moreover, weak genotoxic effects on the colon of rats treated with NM-203 (5–10 and 20 mg/kg for 3 days by oral gavage) were reported only at the lowest dose (5 mg/kg) [37]. However, the authors conclude that further investigations are requested to establish the genotoxicity of NM-203 [37]. When two mesoporous silica NFs were tested (size of 100 and 300 nm) for 5 consecutive days by oral gavage, Cabellos and co-authors showed no relevant effects pertaining the genotoxic effects on intestinal barrier [40]. In summary, the reported Tier 3 existing data are limited, though they provide sufficient evidence on the suitability of the Tier 1 and Tier 2 data for measuring local effects in the intestine upon repeated exposure. The similarity assessment of Tier 1 and Tier 2 data indicates that read-across may be possible from the NFs that possess Tier 3 data to those that lack Tier 3 data. However, when a wider variety of in vivo data on source materials will become available further case studies to validate the IATA will be useful.

### Expert judgement to accept or reject hypothesis H–O–G1 and conduct read-across for the case study silica NFs

The results obtained for the Tier 1 and Tier 2 hazard data, for the OGI fluid dissolution and hazard DNs, support the similarity of the silica NFs used in this study. It appears that the silica NFs are similar and show low toxicity (cytotoxicity, barrier integrity and inflammation) toward intestinal cells (Tier 1 and 2) at the experimental conditions implemented. However, Tier 2 model increases the confidence level of similarity of the silica NFs to each other in terms of their lack of ability to induce production of the pro-inflammatory mediator. The collected in vivo Tier 3 data, although limited, confirm such a thesis, making possible the read-across. Thus, we conclude that the tested silica NFs can be grouped based on the oral IATA and in case of testing of other target SiO_2_ materials, we recommend reducing the testing (especially when precautionary is the reason for grouping).

Accordingly to recent scientific evidence [21], the TTS recognizes the importance of dosimetry assessment (e.g. measuring the nominal vs. effective dose as well as the sedimentation rate of NFs and/or effective cellular uptake) when evaluating the toxicity of high dense NFs by in vitro Tier 1 and Tier 2 methods. In our case study, as low density and stably suspended NFs are involved, the issue of sedimentation as well as the calculation of the effective dose is not relevant. Indeed, the results obtained using the nominal dose at both Tier 1 and 2 levels align well with Tier 3 in vivo data indicating absence of local toxicity for SiO_2_ NFs.

Moreover, the wording of the original hypothesis H–O-G1 could be made more specific. The original hypothesis suggests that gradual dissolving NFs may be associated with local toxicity of the OGI tract driven by the NF or the ions/molecules released (Additional file 1: Figure SI1). The results however indicate low toxicity to the OGI tract for all of the silica NFs and literature based evidences highlight the inherent biocompatibility of the silicates ions. The following grouping hypothesis that aligns with the data would therefore be more specific and more relevant to the specific scenario investigated in this case study: *Following ingestion of amorphous and mesoporous silica NFs, which are gradually dissolving in OGI fluids, such NFs will not induce local toxicity to the gastrointestinal tract upon repeated exposure*”. For the NFs presented, in vivo local toxicity data was available only for NM-203 (Table 3). Since the NFs can be successfully grouped according to the modified hypothesis (all exhibiting similarly low toxicity in vitro), it is possible to use read-across to conclude they do not likely induce intestinal histopathological changes in vivo.

### DN addressing systemic tissue hazard of NFs (H–O–G2): data generation and interpretation

Since silica NFs are grouped as gradually dissolving according to OGI and PSF fluid dissolution kinetics, they have the potential to translocate to secondary target organs leading to systemic toxicity. For NFs that, owing to biopersistence, have the potential for accumulation at any level, the IATA allows investigation for systemic toxicity.

The grouping hypothesis that addresses accumulation assumes the potential for NFs to reach secondary organs, with the liver considered the main target organ of NF toxicity (Additional file 1: Figure SI1) [48, 49]. Tier 1 data was therefore produced using a 2D liver in vitro model, the hepatocyte cells HepG2/C3A cells, which are widely used for assessing the NM toxicity to the liver [50]. Two endpoints were measured: the cytotoxicity and the inflammatory response (IL-8 gene expression and the corresponding protein secretion). Cells were treated for 24 h with all silica NFs using three representative concentrations (9.6, 48 and 96 ug/mL) as selected for the intestinal models at Tier 1. The MCM-60 and MCM-170 produced highly similar responses in local toxicity experiments (Fig. 4) and so only MCM-60 was used in the following study.

**Fig. 5:**
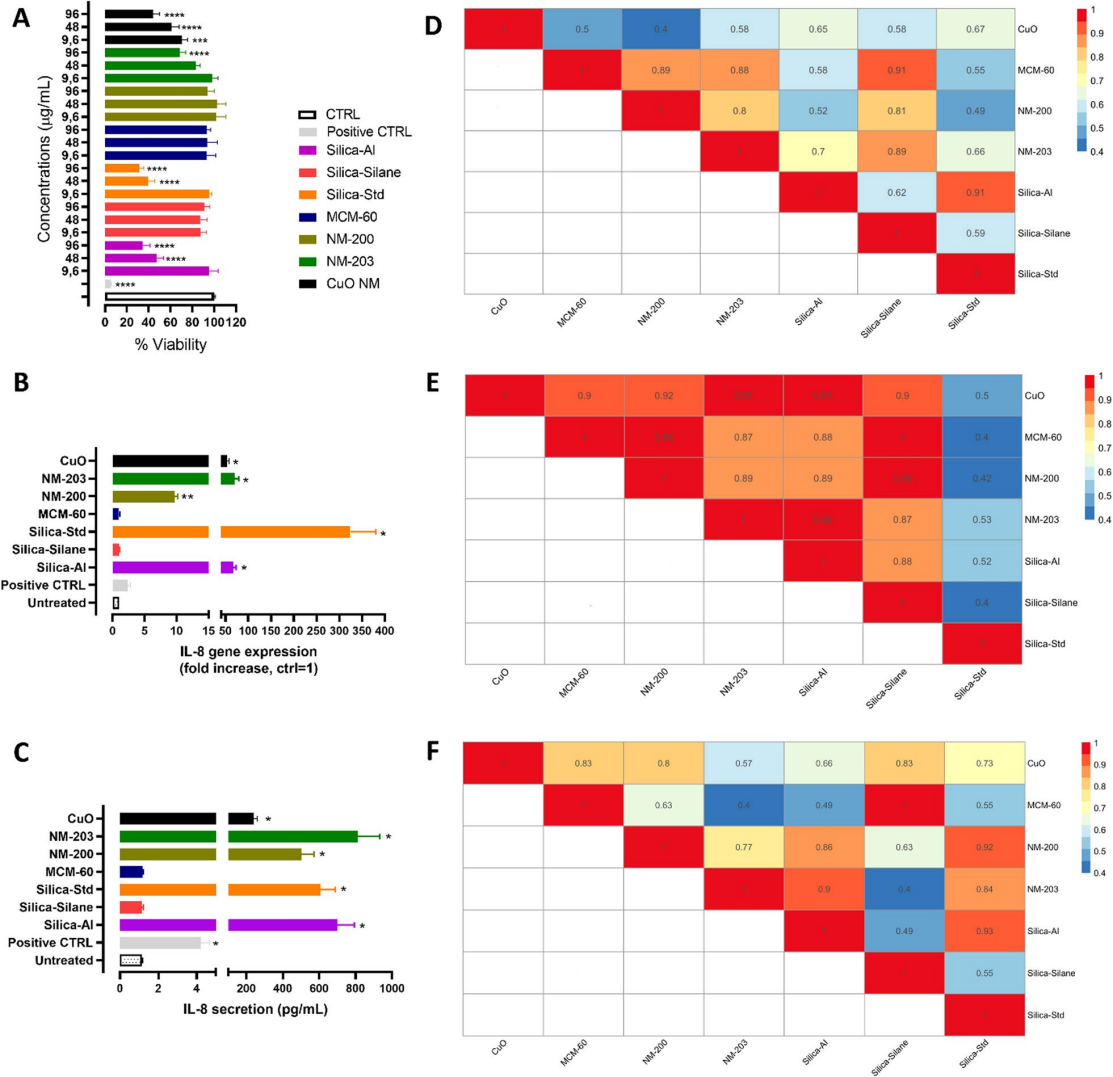
Impact of silica NFs on the in vitro liver model. **A** HepG2/C3A cells were cultured as a monolayer in 96 well plates for 24 h and exposed to 9.6, 48 and 96 ug/mL of Silica and CuO NFs for 24 h followed by the assessment of viability using Alamar blue assay. **B** HepG2/C3A IL-8 expression and **C** IL-8 secretion assessed by real time PCR and ELISA assay, respectively, after 24 h of exposure to NFs (48 µg/mL). **D**, **E** and **F** BF pairwise similarity assessment was conducting comparing the curves of % viability, HepG2/C3A cells IL-8 mRNA expression fold increase and IL8 protein production measured in pg/mL, together with the concentration values. BF Values range between 0 and 1 (similar NFs). BF Values close to zero indicate that the NFs are not similar. Data are shown as mean ± standard deviation. *p < 0.05, **p < 0.01, ***p < 0.001 and ****p < 0.0001 versus untreated. Triton X100 (0.01%) and LPS (10 µg/mL) were used as positive control for viability and cytokine expression/secretion, respectively

Figure 5A shows a dose-dependent significant toxicity of the positive control CuO NM, which was similar in magnitude to the effects induced by Silica-Al and Silica-Std. NM-203, also induced a dose-dependent significant cytotoxicity reaching 70% viability at the highest exposure concentration. The other silica NFs (MCM-60, NM-200 and Silica-Silane) did not show any significant viability impairment at the experimental conditions applied.

The inflammatory response was assessed by IL-8 expression (Fig. 5B-C). Figure 5B reports IL-8 mRNA expression after 24 h incubation of HEPG2/C3A with 48 µg/mL of the tested silica NFs, while Fig. 5C represents the IL-8 protein data. In line with the cytotoxicity data, both IL-8 mRNA and protein were significantly induced by Silica-Al, Silica-Std and NM-203, but not by MCM-60 and Silica-Silane. NM-200, which did not induce cytotoxicity, did however demonstrate an increase of IL-8 mRNA and protein expression.

Interestingly, the HepG2 cellular model is demonstrated to be more susceptible to the NF insult compared with the intestinal epithelium models, the Caco-2 models. Similar evidences are indeed reported also for other NMs such as silver NM [51] and food grade nanoemulsions [52] showing highest toxicity to liver cells than intestinal cells.

### DN addressing systemic tissue hazard of NFs (H–O–G2): similarity assessment.

The similarity assessment based on BF calculation was performed on the tested biological descriptors (Fig. 5D, E and F) related to systemic toxicity. The analysis of the viability data firstly has shown that the silica NFs do not form a single group. Instead, two groups are identified, the first is formed by MCM-60, Silica-Silane, NM203 and NM-200, while the second is formed by the Silica-Std and Silica-Al (Fig. 5D, Table 4).

**Table 4:** Summary of the groups generated by the similarity assessment on the HepG2/C3A hazard in vitro assays related to the H-O-G2 hypothesis

Viability grouping		IL-8 mRNA grouping		IL-8 Protein grouping	
Group 1	Group 2	Group 1	Group 2	Group 1	Group 2
MCM-60 Silica-Silane NM-200 NM-203	Silica-Std Silica-Al	MCM-60 Silica-Silane NM-200 NM-203 Silica-Al	Silica-Std	MCM-60 Silica-Silane	Silica-Std Silica-Al NM-200 NM-203

The inflammation data (measured as IL-8 mRNA expression level) demonstrate two different groups where MCM-60, Silica-Silane, NM203 and NM-200 plus Silica-Al represent a group and the Silica-Std alone represent the other group (Fig. 5E, Table 4). The results were different for the IL-8 protein data, with the MCM-60 grouping with only the Silica-Silane, but not with the NM-203 or NM-200, which are, instead, grouped with Silica-Al and Silica-Std (Fig. 5E, Table 4). Finally, the behavior of silica NFs compared to CuO NM is interesting. Indeed, the silica NFs panel appear similar to CuO for some endpoints and dissimilar for others. For example, when the inflammation DN (at mRNA level) (Fig. 5E) is considered, silica NFs and CuO NM are similar, while when the viability DN is considered (Fig. 5B) they are not similar. This data could be explained as the CuO NM does not belong to the same dissolution group of silica NFs for the PSF fluids (Fig. 3A). In fact, CuO NM dissolves faster and accordingly to the oral IATA cut-offs (Fig. 1) this material belongs to the “quick dissolving group”. Furthermore, Cu^2+^ ions are more toxic than silicon ions, thus toxicity derived from ions within the cells is more relevant and therefore CuO NMs could follow a different mechanism of toxicity.

To conclude, the similarity assessment performed on Tier 1 hazard data highlights the presence of many different groups of silica NFs, that cannot be referred to the groups generated by the dissolution in PSF (colloidal silicas *vs*. NM-200 and NM-203), demonstrating that the systemic toxicity induced by silica NFs should be investigated by data at higher level of confidence (i.e., Tier 2), or on a case by case basis.

### Existing Tier 3 in vivo data required to test the H–O–G2 hypothesis

In vivo (Tier 3 level) data from the literature (reported in Table 3) were analyzed to fulfil the TTS and to provide scientific based evidence to calibrate the Tier 1 results of the H–O–G2.

The available in vivo data for the tested silica NFs is available in three publications, where rats or mice were exposed via oral intake to NM-200 and NM-203 (established as source materials for the case study) for short [37] and long time periods [36, 41]. For the short time period, Tarantini et al. evaluated liver, spleen and kidneys histopathologically without finding any toxicity after a 3-times repeated exposure to 5, 10, or 20 mg/kg bw of NM-203 [37]. For the longer time periods Tassinari et al. and Boudard et al. reported silicon ion accumulation (total silicon content) in the liver leading to inflammatory events after sub-chronic and chronic exposure to doses of NFs with a physiological relevance for human intake (2–5 mg/kg bw) [36, 41]. In particular, Boudard et al. reported that liver inflammation resulted in amyloidosis in liver perivascular regions of mice treated with NM-200 via drinking water for 18 months (4.8 mg/kg bw) [41]. Moreover, the authors also showed histomorphological findings in the mouse kidneys consisting of vacuolization of tubular epithelial cells. On the other hand, Tassinari et al. documented hepatic inflammation leading to enlarged liver sinusoids in rats treated via oral gavage to NM-203 for 90 days (2–50 mg/kg/bw) [42]. In this work, there was also evidence of histomorphometrical alterations in the spleen and changes in blood immunotoxicity markers.

Two studies are available on the investigation of systemic genotoxic effects possibly induced by NM-203 [45, 46]. In both studies rats were orally treated with 2, 5, 10, 20, or 50 mg/kg for 90 days. Villani et al. show that the spleen appears to be the only target organ of NM-203, showing a weak genotoxic effect. However, in this case, the authors conclude that further studies are required to confirm a clear genotoxicity possibly induced by NM-203 in splenocytes [45]. On the other side, Tassinari and co-authors focused their study on the effects posed by NM-203 on the rats’ reproductive systems finding no genotoxicity on reproductive organs of male and female rats [46].

Studies utilizing the intravenous route of exposure are of course considered relevant only for drug administration and not as involuntary route for human exposure as oral ingestion may be. However, they may provide here some information related to systemic toxicity. Intravenous injection of a higher dose of NM-203 (20 mg/kg/bw) in rats for either 1 day or 5-repeated days induced chronic inflammatory (granulomas) lesions in the liver 90 days after the exposure, although this differed with sex [42]. Indeed, for males these granulomas appeared after a single and 5-day exposure, while for females, lesions appeared only after the 5-day repeated exposure. Moreover, an evident splenomegaly accompanied by the presence of inflammatory infiltrates consisting of both mononuclear cells and granulomas was reported [42].

Systemic toxicity was also assessed for colloidal silicas. Interestingly, Buesen et al. reported no adverse systemic findings in rats under the reported experimental conditions (28-days oral exposure up to and including 1000 mg/kg/bw of four different Levasil® 200 NFs, one of which is comparable to our Silica_Std) [39].

An in vivo study on the MCM silica NFs (100 and 300 nm; 10–844 m^2^/g) which involve a short-term oral exposure (5 consecutive days of oral gavage administration to doses of 100 to 1000 mg/kg/bw) showed no relevant systemic effects (including genotoxicity) along with no relevant alteration of the liver functions [40], although the results by a hyperspectral imaging microscopy system analyses suggested the presence of silica particles in the intestinal tissue of exposed animals [40]. However, opposite results were found by Deng and co-workers when a more prolonged exposure was applied in mice (two weeks instead of 5 days of 50–100-200 mg/kg/bw of MCM silica NFs 75 nm and with an SSA of around 40,000 m^2^/g) by oral gavage. They indeed found a significant enhancement of the levels of serum alkaline phosphatase (ALP), aspartate aminotransferase (AST) and alanine aminotransferase (ALT), infiltration of inflammatory cells in the spleen and intestines. Interestingly, as for the work of Cabellos and co-workers[40], they found in the treated animals a higher total silicon content in many secondary organs (e.g. liver, spleen, lung, and kidney) with respect the controls, thus suggesting an accumulation during the entire treatment. Further, a TEM analysis on intestinal tissue revealed the distribution of small dense dots likely referring to agglomerated particles, suggesting that the detected silicon content may be in part referred to intact particles, which deposit upon the absorption (although the presence of silicon ions cannot be excluded by the presented data) [43]. The systemic toxicity as shown by this work, however, refer to MCM silica NFs highly different in term of SSA with respect the MCM used in this work.

In addition, a study investigating the acute toxicity following a single dose (100 mg/kg) of colloidal (46 and 432 nm) and mesoporous silica NFs (466 nm) by intravenous administration in mice, showed a significant amount of liver inflammation and other tissue injury in several organs (heart, lungs, spleen and kidney). The observed pathologic lesions were mostly noted in the animals injected with large colloidal silica NFs compared to small colloidal and mesoporous silica NFs [44]. The authors demonstrated that it took one year for the animals to recover from acute toxicity of silica NFs following a single intravenous dose.

Finally, it may be worth mentioning that the risk assessment of oral exposure to amorphous silica NFs present in foods remains greatly uncertain due to technical drawbacks (silicon detection, contaminants, exposure mode) that must still be solved [53].

In summary, the few reported Tier 3 data on systemic toxicity is not sufficient to conclude whether the Tier 1 testing is suitable to support assessment of similarity and grouping of the silica NFs.

### Expert judgement to accept or reject hypothesis H–O-G2 and conduct read-across for the case study silica NFs

The results obtained for the Tier 1 data refer to the PSF fluid dissolution and hazard DNs do not support the similarity of systemic hazard of the silica NFs used in this study, as different grouping of silica NFs was evident depending on the endpoints tested. Indeed, the different in vitro assays, selected for the hazard DNs, were not consistent in how the NFs group in terms of the ability to induce cytotoxicity, IL8 mRNA or IL8 protein production (Table 4). Such inconsistency in the silica NF in vitro toxicity data prevents their grouping and suggests that for this case study, it is not possible to use the few in vivo systemic data for NM-200 and NM-203 to support read-across and fill the hazard data gaps for the remaining silica NFs. Furthermore, there is no relationship between the groups formed by the PSF dissolution DN and the systemic hazard DNs. For this reason, a similarity assessment and read-across could not be conducted for this data set.

The conclusion is therefore that we cannot group the NFs using the H–O–G2 hypothesis because the NFs are not sufficiently similar to each other to be grouped, according to the pairwise BF analysis threshold values. The in vivo data available in the peer reviewed literature suggests that NM-200 and NM-203 can translocate from the gut to the liver following treatment at dose levels that were selected to be as close as possible to the expected human exposure to silicon dioxide [36, 41]. For this reason, to verify the hypothesis, we would recommend increasing the confidence level of the data by using the Tier 2 testing. The Tier 2 testing includes the use of advanced and more physiologically relevant in vitro models (i.e., human hepatocytes, liver spheroids, etc.) along with experimental conditions, such as repeated exposures and verification of chronic responses which may be more relevant conditions to tackle a systemic toxicity induced by accumulation of NFs [54]. Finally, with the increasing of in vivo studies, in the future, we expect the potential application of read-across by using Tier 2 data.

### Discussion

The first aim of this study was to assess whether pre-defined oral grouping hypotheses (H–O–G1 and H–O–G2) and corresponding IATAs could be used to support grouping of silica NFs:

For the H–O–G1 which relates to the ability of gradually dissolving NFs to induce local toxicity in the OGI tract, the oral IATA (and relative TTS) worked effectively to support grouping. The similarity assessment at both Tier 1 and 2 level supported the grouping hypothesis. In particular, the Tier 1 data (including the cascade dissolution in vitro assay, cellular reactivity, cell viability, membrane damage and cytokine release) (Table 1) suggested similarity between the different NFs but with some small variations. Interestingly, these variations disappeared when the Tier 2 method was implemented next. These results suggested that the Tier 2 information was capable in enhancing the level of confidence so that all NFs were considered similar with the highest level of similarity score available (Fig. 3).

Moreover, as the Tier 3 data on source material is in accordance with Tier 1 and Tier 2 data, a read-across was possible across them. Read-across of in vivo data from the source materials (NM-200 and NM-203) to the target NFs (Silica-Silane, Silica-Std, Silica-Al, MCM-60 and MCM-170) was therefore conducted, and we were able to conclude that the group members are all unlikely to induce histopathological changes in gastrointestinal tissues following repeated exposure to the silica NFs by ingestion.

By the application of the oral IATAs, we recommend to focus on Tier 2 methods as they gave a clearer similarity assessment especially when the grouping decision is taken for regulatory reasons. The effectiveness of the IATAs although demonstrated on negative data *e.g.* lack of toxicity for SiO2 NFs is proven by the positive control, the CuO NFs for which Tier 1 and 2 demonstrated instead toxicity. These data were also confirmed by Tier 3 in vivo evidences.

Lastly, while the data generated did support the grouping of the NFs, it was possible to make more specific the wording of the original local toxicity (H–O–G1) hypothesis. This process therefore went beyond the original aim, demonstrating how the data generated via the IATA can be used to reformulate the hypothesis (in line with the GRACIOUS Framework) and make it more appropriate and more specific to the NFs investigated.

For the systemic toxicity (H–O–G2), however, the patterns of similarity across the Tier 1 data were inconsistent for the different tested DNs (both dissolution and hazard DNs). For this hypothesis Tier 3 data is limited although studies report that a systemic toxicity for silica NFs can be likely. Based on the application of the oral IATA using only Tier 1 data neither grouping nor read-across was appropriate due to a lack of similarity. For such a case study, we recommend moving forward through the oral IATA applying the Tier 2 level of testing. We expect that by the inclusion of Tier 2 data, the confidence level of the data will improve. This approach might allow for the production of high content data possibly comparable with the in vivo data (already available or newly coming) in a read-across exercise. However, it is possible that grouping for these NFs may not be possible with respect to systemic effects, leading to the requirement for a case-by-case assessment.

Another aim was to apply different methods for assessing similarity to support a quantitative similarity assessment. The details of these methods are described elsewhere [9, 26] but were applied here to demonstrate their usefulness. The data provided by the case study allowed for the testing of two approaches the BF and the clustering analyses. Results confirmed that these tools are effective to identify similarities among different NFs and for different biological and dissolution endpoints.

Overall, we have demonstrated the importance of testing the similarity of different NFs by the combination of the different DNs in an IATA to reach a grouping decision. The IATA facilitated the structured data gathering and, by their interpretation, supported read-across with the available in vivo data. The IATAs also support the users in taking specific actions, for example, in reducing the testing by suggesting specific questions to address regarding the toxicity of the NFs for which the grouping is requested or moving forward to advanced testing to obtain more focused information. Similar works testing other developed GRACIUOS IATAs support the evidence presented in this study. For example, Braakhuis et al. have recently developed an inhalation IATA [7] that was tested using 16 organic pigments and 4 representative test materials. It was demonstrated that the IATA delivered consistent groupings through the assessing of Tier 1 methods which were able to exclude some candidate NFs from the group, but also to suggest limits of acceptable similarity for others [55]. Moreover, when Tier escalation was applied (from Tier 1 to Tier 3) for some candidate NFs, the conservative group established by the Tier 1 data was demonstrated [55]. Another work includes the testing of the high aspect ratio nanomaterials (HARNs) IATA [5]. Murphy and co-authors tested 15 different multiwalled carbon nanotubes (MWCNT) demonstrating the successful application of the HARN IATA to group some MWCNT with a similar hazard potential after inhalation exposure, while discriminating from others without a similar inhalation hazard [56].

## Conclusions

The hypothesis describing the potential for local toxicity to the OGI tract could easily be assessed by applying the IATA. Further the case study indicates the strength of the oral IATA by allowing for an improvement in specificity of the hypothesis wording to better address local hazard effects. Moreover, it excludes the need of additional rodent tests, recommending the use of Tier 2 testing that provides a clearer similarity assessment. The hypothesis describing systemic effects was more ambiguous in terms of conclusions and interpretations. There was a lack of similarity of the in vitro results and the availability of in vivo data was insufficient. Therefore, the recommendation is to apply more advanced testing such as Tier 2 and/or in vivo Tier3 in order to make the oral IATA related to systemic toxicity much more exploitable and useful for grouping and read-across.

In conclusion, the testing of the oral IATAs has demonstrated its suitability for supporting grouping of NFs for similarity assessment and for read-across of hazard data from source NFs to target NFs. The grouping and similarity methods were sufficiently robust to identify sub-groups, to remove NFs from a group and to reject grouping hypotheses when appropriate. Overall, the different similarity algorithms generated comparable responses. However, when insufficient or diverse data were available, grouping conclusions were also diverse indicating the need to incorporate extra information in the TTS and further develop them. Indeed, with the future progress of the characterization techniques and their incorporation in the TTS of oral IATAs, a reduction of the data uncertainty and a better data integration (to improve the expert interpretation of the similarity outcomes) are expected.

## Materials and methods

### Chemicals and reagents

All chemicals and reagents used were obtained from Sigma-Aldrich, unless otherwise stated.

### NFs and their physical chemical characterization by transmission electron microscopy (TEM) and dynamic light scattering (DLS)

Synthetic amorphous silicon dioxide NFs (SiO_2_, SAS), the JRCNM02000a and JRCNM02003a, were obtained from the JRC Nanomaterials Repository (Ispra, Varese, Italy) and from now they will be indicated as NM-200 and NM-203, respectively. Silica-Std, Silica-Al and Silica-Silane NFs are commercial colloidal amorphous silicas provided to the GRACIOUS consortium by Nouryon (Sweden). Mesoporous silica nanoparticles of 60 and 170 nm size (referred to as MCM-60 and MCM-170, respectively) were produced by the so-called liquid–crystal-templating mechanism, following the protocol reported by Catalano et al.[20]. The MCM-170, although not a NF as it is composed of particles with a size greater than 100 nm, was included in the study as it is possible that the interaction with body fluids may generate nanoparticles smaller than 100 nm.

Copper oxide (CuO) NFs was introduced in this study as positive control to test the hazard DNs as it is a well- known toxic nanomaterial [35]. This NF was purchased from Sigma Aldrich (Milan, Italy), and Plasma Chem, GmbH (Berlin, Germany).

Before the experiments, NM-200, NM-203 and CuO NFs were heated at 230 °C for 4 h to eliminate possible contamination from lipopolysaccharide (LPS) [57, 58]. For the other NFs, as they were already provided as dispersion by the suppliers, they were tested for the presence of endotoxin by Limulus Amebocyte Lysate (LAL) assay (Pierce, Thermo Scientific, Italy). The obtained values of endotoxin were below the limit of 0.5 EU/mL as required by the US Food and Drug Administration (FDA) guidelines [59].

NM-200 and NM-203 were provided in powders. Stock suspensions in Milli-Q® water (2.56 mg/mL) were sonicated using a Bandelin Sonopuls Ultrasonic Homogenizer HD 2200, equipped with a 3-mm probe (BANDELIN electronic GmbH & Co. KG; Berlin, Germany). An amplitude to about 30% of the maximum (302 µm) was applied for 5 min to gently favor the nanoparticles dispersion in the suspension. Before diluting, the stock suspensions were left to equilibrate at room temperature for 30 min.

Silica-Std, Silica-Al, Silica-Silane, MCM-60 and MCM-170 were provided in water (at 300 mg/mL, 250 mg/mL, 280 mg/mL, 7.3 mg/mL and 7.3 mg/mL, respectively) and sonicated for 10 min by a Bandelin Sonorex Ultrasonic Bath (BANDELIN electronic GmbH & Co. KG; Berlin, Germany), as indicated by the manufacturers.

The morphology of all NFs was evaluated by TEM using JEOL JEM-1011 (Jeol Ltd.; Akishima, Japan), equipped with a W thermionic source operating at 100 kV. For NM-200, NM-203, MCM-60 and MCM-170, the stock suspensions were diluted 1:10 in minimum essential medium (MEM) supplemented with 2 mM L-glutamine (selected here as representative cell culture medium) or Milli-Q® water. For Silica-Std, Silica-Al, Silica-Silane, the stock suspensions were diluted 1:500 in both the dispersants. For each diluted working suspension, 5 µL was dropped onto a carbon-coated copper grid (Electron Microscopy Sciences; Hatfield, PA, USA) and were left to air dry.

DLS analysis by Malvern Zetasizer Nano-ZS (Malvern, UK) was used to measure the size distribution profiles of NFs in both the dispersants. Size profiles of NFs were monitored at time zero (t_0_), and over 24 h according to the timing of the employed in vitro cellular experiments. For the analysis, an appropriate volume of each sample was added to 1 mL of the dispersants to reach the desired working concentration (96 µg/mL) and analyzed using disposable polystyrene cuvettes at 37 °C. Consecutive measurements were acquired at different time points (0, 1, 2, 4, 6, 8 and 24 h, 10 repeated measurements for each point). The optical indices of the instrument (Ri and Rabs) were set to 1.544 and 0.20 for all the silica NFs, according to NanoREG SOP “*NRCWE SOP for measurement of hydrodynamic Size-Distribution and Dispersion Stability by Dynamic Light Scattering (DLS)*”. Results were expressed as hydrodynamic diameter (D_H_).

### Cascade in vitro digestion assay

The in vitro digestion assay was performed following the protocol from our previous works [22–24]. Simulant body juices (saliva, bile, and from stomach and duodenum) were prepared (w/o the proteins and enzymes) on the day prior to their use and incubated overnight at 4 °C. The day of the experiment the completed juices (w proteins and enzymes) were then heated to 37 °C for 2 h before use. The pH of saliva, stomach, duodenum and bile fluids has to be 6.8 ± 0.1, 1.3 ± 0.1, 8.1 ± 0.1 and 8.2 ± 0.1, respectively. Silica sample handlings were carried out in a way to minimize silicon background (silicon release from equipment) using ultrapure reagents and disposable sterile polypropylene tubes. The digestion process, conducted at 37 °C and under shaking at 80 RPM, started when 2 mL of saliva juice was added into a Falcon tube to 0.3 mL of NFs at the working concentration of 1 mg/mL. After 5 min, 4 mL of stomach juice was added to the resulting solution for another 120 min (pH adjusted to 2.5 ± 0.5). A solution of 4 mL of duodenal fluid, 2 mL of bile salts and 0.6 mL of sodium bicarbonate (84.7 g/L) was finally added for another 120 min to complete the in vitro digestion under intestinal simulating conditions (pH adjusted to 6.5 ± 0.5). For post-digestion analysis, samples were collected after 30 min of incubation into the intestinal fluid incubation (which is after the first 155 min of total assay time) as this time is physiological relevant for intestinal adsorption [25]. The free ion fraction of digested NFs was separated by Ultrafiltration (UF) (15 mL Amicon Ultra centrifugal 3 K filters, Millipore) and quantified by Inductively Coupled Optical Emission Spectrometry (ICP-OES, Agilent 720/730 spectrometer). The dissolution rate of NFs was calculated as half-time (t_1/2_) and as percentage (%) of dissolution. The calculation of the half-time is assessed according to Keller et al. and Di Cristo et al. [6, 60] as it follows:

The total ion mass dissolved at time t [Mion (t)] obtained by ICP analysis is used to derive the dissolution k rate (ng/cm^2^/h) as follows:

1 $$K_{dis} = \frac{{M_{ion} \left( t \right)}}{SA \left( t \right)}/\Delta t$$

where ∆t is the sampling interval time (155 min) and SA (t) is the total surface area at time t and is approximated as:

2 $$SA\left( t \right) = BET\left( {t_{0} } \right)*\left( {M_{0} - M_{ion } \left( t \right)} \right)$$

SA is obtained by multiplying the Brunauer–Emmett–Teller (BET) value at time 0 (t0) to the M_0_-Mion(t), where M_0_ is the total ion mass of the NF at time 0, supposing a 100% dissolution (taking into account the compartment dilution factor during addition of different media (i.e., 1:39 for the intestine)).

K_dis_ can be converted to dissolution half-time t_1/2_ (h) by:

3 $$t_{1/2} = \frac{\ln \left( 2 \right)}{{BET \left( {t0} \right)*K_{dis} }}$$

The calculation of the % of dissolution is performed as follows:

4 $$\% dissolution \left( t \right) = \frac{{M_{ion} \left( t \right)}}{{M_{0} }}x 100$$

### In vitro dissolution assay in phagolysosomal (PSF) fluid

The experimental protocol for assessing dissolution was recently described in Keller et al. (2021a). The “continuous flow system” (CFS) is an adapted version of a dissolution set-up for man-made vitreous fibers (MMVF) [61] adjusted to the small pore sizes required for NFs. This adapted set-up is in agreement with ISO/TR19057:2017 and describes the dissolution rate as well as the half-time. In contrast to alternatives such as dialysis bags [62], agglomeration and settling is no concern because the NFs are held between two horizontal membranes, and constantly flushed by medium. Overall, the set-up consists of three major components: the flow-through cell, the medium and the technique for quantification of the dissolved fraction. For each material, 1 mg was transferred onto a 5 kDa cellulose triacetate membrane (Sartorius Stedim Biotech GmbH, Goettingen, Germany) and inserted in the flow-through cell, onto which was placed another 0.45 µm membrane. The elution medium was PSF at pH4.5, which was developed by NIOSH [63] and is recommended to investigate the dissolution under lysosomal conditions by the ISO standard (ISO/TR19057:2017). The flow cells were temperature-controlled at 37 ± 0.5 °C. By comparison to in vivo clearance of titanium dioxide (TiO_2_), zinc oxide (ZnO) and barium sulfate (BaSO_4_) NFs, representing very slow, quick and gradual dissolution respectively, we had previously calibrated the flow rate to 2 mL/h for best predictivity [60]. Over a duration of 7 days, eluate samples were analyzed for the Si ion concentration through ICP-OES. The time-resolved mass values were added together and corrected for SiO_2_ stoichiometry to construct the dissolution kinetics. Dissolution rates are determined for each sampling step as described in paragraph 2.3.

### Acellular reactivity by Dichlorodihydrofluorescein diacetate (DCFH_2_-DA) assay

Detection of reactive oxygen species (ROS) production using the 2′-7′-dichlorodihydrofluorescin diacetate (DCFH_2_-DA) assay was conducted as follows. DCFH_2_-DA was chemically hydrolyzed by incubation with 0.01 M NaOH, neutralized and diluted to 10 µM DCFH_2_ in phosphate-buffered saline (PBS). During this reaction, test particles were prepared by suspension in phenol red-free minimum essential medium (MEM) with 2% fetal calf serum (FCS) at a concentration of 1000 µg/mL, followed by ultra-sonication in a water bath and serial dilutions to obtain a range of 156–1000 µg/mL. Each treatment was then added, in triplicate to a 96-well plate at a volume of 25 µl, followed by addition of 225 µl 10 µM DCFH_2_ to each well. Final concentrations of 1.56–100 µg/mL were obtained, which were incubated at 37 °C for 90 min. The CuO NM (1.56–12.5 µg/mL) was included as an oxidant-producing, positive control for the assay. After 90 min, samples were centrifuged at 3000 × g for 15 min, and 100 µl of each well was transferred to a black 96-well plate to read fluorescence at ex/em wavelengths of 485/530 nm. To address potential for interference of particles with the light detection, the same process as above was replicated using particles suspended in solutions of PBS alone (no DCFH_2_), or with 0.1 µM fluorescein diacetate (FDA). No interference with fluorescein signal by the particles was detected. To account for background interference, signals generated with incubation in solutions of PBS alone were removed from signals generated in solutions of DCFH_2_.

### Intestine cell culture models

The HT29-MTX clone E-12 cell line was purchased from the European Collection of Authentic Cell Culture (ECACC) (UK) [64] and the human colon colorectal adenocarcinoma Caco-2 cell line (Caco-2 ATCC® HTB-37™) was sourced from the American Type Culture Collection (ATCC) (USA). The Human Burkitti's lymphoma, Raji cell line were obtained from the DSMZ-German Collection of Microorganisms and Cell Cultures GmbH (Germany) [65]. Caco-2 and HT29-MTX clone E-12 cells were maintained in Dulbecco’s Modified Eagle’s Medium—high glucose (DMEM) (Gibco Life Technologies) supplemented with 10% heat inactivated fetal bovine serum (FBS) (Gibco Life Technologies), 100 IU/mL non-essential amino acid (NEAA) (Gibco Life Technologies) and 100 U/mL Penicillin/Streptomycin (Gibco Life Technologies) (termed complete cell culture medium), at 37 °C and 5% CO_2_ and 95% humidity. Caco-2 and HT29-MTX were split twice a week and cells at passage 50 to 60 were used for the experiment. Raji cells were maintained in Roswell Park Memorial Institute (RPMI) 1640 Medium (Gibco Life Technologies) supplemented with 10% heat inactivated FBS (Gibco Life Technologies), 100 U/mL Penicillin/Streptomycin (Gibco Life Technologies) and at 37 °C, 5% CO_2_ and 95% humidity. Raji cells were split twice a week and cells at passage numbers 10 to 15 were used for the study. The intestinal triple culture model was cultured following the method described previously [66] and modified. Briefly, following the addition of 1.5 mL complete cell culture medium at the basolateral (BL) chamber of 3.0 µm pore polycarbonate transwell inserts in a 12-well plate with growth area of 1.12 cm^2^ (Costar corning, Flintshire, UK), 4 × 10^5^ cells of Caco-2 and HT29-MTX cells suspended in 0.5 mL complete cell culture medium were seeded at apical (AP) chamber at the ratio of 9:1. The cells were maintained at 37 °C and 5% CO_2_ and 95% humidity and the medium changed at the AP and BL chamber every other day. On the 16th day, 5 × 10^5^ cells of Raji cells were suspended in 1.5 mL of complete cell culture medium were seeded into the BL chamber and maintained for 5 days at 37 °C and 5% CO_2_ and 95% humidity with daily medium replacement at the AP chamber. The transepithelial electrical resistance (TEER) was measured twice a week (see below) starting from the 10th day to monitor the development of intact barrier integrity. Triple culture with TEER value of 500 Ω cm^2^ and above were used for further experimentation.

### Liver cell culture model and treatment

The human liver epithelial cells, HepG2/C3A, were obtained from the American Tissue Culture Collection (ATCC®) (LG Standards, England). Cells were maintained in Minimum Essential Media (MEM)-supplemented with 10% FBS, 100 U/mL Penicillin/Streptomycin and 2 mM of L-glutamine and were routinely cultured in a humidified atmosphere of 5% CO_2_ in air in T75 cell culture flasks (Nunc, Fisher Scientific, Italy).

### Alamar blue assay

For the intestinal model a concentration of 1.56 × 10^5^ cell/cm^2^ of Caco-2 cells were seeded in 96 well plate (surface are 0.32 cm^2^) (Coaster Corning Flintshire, UK) and maintained at 37 °C and 5% CO_2_ for 24 h. The cell culture medium was aspirated, and cells were washed with PBS (Gibco Life Technologies) two times. The cells were then exposed to 100 µl of culture medium (negative control), various concentrations of NFs ranging from 0.98 to 125 µg/mL and 0.01% Triton X100 (positive control). Following 24 h incubation, the supernatants were removed and stored in − 80 °C freezer. The cells were washed twice with PBS and 0.1 mg/mL (100 µl) Alamar blue reagent Sigma (Poole, UK) diluted in cell culture medium were added into each of the wells and incubated for 4 h at standard cell culture condition. The fluorescence readings were taken with a microplate reader, SpectraMax M5 (California USA) and the results were presented as mean % viability ± standard deviation.

For the liver model, a concentration of 3 × 10^4^ cell/well of HepG2/C3A were seeded in a 96 well plate (surface are 0.32 cm^2^) (Coaster Corning Flintshire, UK) and maintained at 37 °C and 5% CO_2_ for 24 h. The cell culture medium was aspirated, and cells, after washing, were then exposed to 100 µl of serum free cell culture medium (negative control), three representative concentrations of NFs (9.6, 48 and 96 µg/mL) and 0.01% Triton X100 (positive control). Following 24 h incubation, the supernatants were removed and stored in − 80 °C freezer. The viability of HepG2/C3A cells was assessed by the Alamar Blue assay as previously described [57, 58, 67]. Cell viability was calculated as a percentage (%) relative to the untreated (negative) control cell cultures. Fluorescence, measured at 572 nm, was performed by means of a Tecan Spark multimode microplate reader (Tecan Italia Srl, Italy).

As NFs could interfere with this assay, a preliminary experiment was performed incubating the dye with diluted NF stock suspension (to reach the dose implemented for the experiments). No fluorescence signal was detected above the background signal (data not shown).

### Cytokines secretion

For the intestinal models, the undifferentiated Caco-2 cells were exposed for 24 h and the supernatant collected and stored in -80 °C freezer, while the triple culture model was exposed to NFs for 5 consecutive days and the fifth day apical supernatant was collected and stored in − 80 °C freezer after 103 h. On the day of cytokine analysis, the supernatant and the reagents were equilibrated to room temperature and the interleukin-8 (IL-8) protein level quantified using Enzyme-linked Immunosorbent Assay (DuoSet ELISA kit) (R&D Systems, Abingdon, UK) following the manufacturers protocol. Tumor necrosis factor alpha (TNF-α, 2 µg/mL) was used as positive control. A TECAN Spark 10 M plate reader (Männedorf, Switzerland) was used to measure the absorbance at the wavelength of 450 nm and the IL-8 level was calculated from the standard curve using 4 parametric logistic fit and presented in pg/mL.

For the liver model, the pro-inflammatory response was investigated after 24 h of treatment by quantifying the accumulated amount IL-8 release in the medium by using the commercially available biolegend ELISA MAX™ Deluxe kits (Campoverde, Italy) according to the supplier’s manual. Lipopolysaccharide (LPS, 10 µg/mL) was employed as a positive control for the induction of the pro-inflammatory response. A Tecan Spark microplate reader was used to detect the optical density at 450 nm. The absorbance at 570 nm was read and subtracted from the absorbance at 450 nm to obtain the corrected (blanked) values.

As NFs could interfere with the ELISA assay by interacting non-specifically with proteins [68, 69], the IL-8 standards were dissolved in the assay diluent (as for manufacturer’s protocol) or in assay diluent spiked with the tested NFs [58]. We have found that CuO and Silica-Al NFs slightly affected the ELISA readouts, quenching the absorbance signal, as shown in Additional file 1: Figure SI9. Hence, cytokines concentrations in supernatants were extrapolated using the ELISA calibration curves of each tested NFs.

### Gene expression analysis

The expression of IL-8 was assessed via quantitative Real-Time PCR (qPCR). At the end of the treatment (24 h), cells were extensively washed with PBS and incubated at − 80 °C with TRIzolTM Reagent (Invitrogen-Thermo Fisher Scientific, Waltham, MA, USA). Total RNA was isolated according to the Chomczynski’s protocol [70] and finally, dissolved in DNase/RNase-free water (Invitrogen-Thermo Fisher Scientific, Waltham, MA, USA). RNA concentration was measured using NanoDrop OneC (Thermo Scientific TM-Thermo Fisher Scientific, Waltham, MA, USA). All samples showed a good purity with A260/A280 ratios of at least 1.7 [71]. SuperScriptTM VILOTMcDNA Synthesis Kit (Invitrogen-Thermo Fisher Scientific, Waltham, MA, USA) was used for the reverse-transcription of total RNA to first-strand cDNA (1.5 μg of RNA per sample in a 20-μL reaction), following manufacturer’s instructions. The qPCR was performed using iTaqTM Universal SYBR® Green Supermix (Bio-Rad, Hercules, CA, USA) on Applied Biosystems ViiA 7 Real-Time PCR System (Life Technologies-Thermo Fisher Scientific, Waltham, MA, USA). Primer sequences used were: forward 5′-CCAGGAAGAAACCACCGGA-3′ and reverse 5′-GAAATCAGGAAGGCTGCCAAG-3′ for IL-8; forward 5′-AAGGTGAAGGTCGGAGTCAA-3′ and reverse 5′-AATGAAGGGGTCATTGATGG-3′ for GAPDH. The GAPDH gene was used as reference gene. For each primer pair, primer specificity was confirmed by melting curve analysis. The quantification of the target transcript relative to the control condition was calculated using Pfaffl’s model [72]. Results are means ± SD of three independent experiments.

### Transepithelial electrical resistance (TEER) measurements

TEER was measured during the development of the intestinal triple culture model and during exposure to NFs. TEER was measured using an epithelial voltohmmeter EVOM2 (World precision instrument, Sarasota, USA), by inserting the long electrode into the BL chamber and the short one into the AP chamber, making sure that the electrode did not disrupt the cell monolayer. The resistance was recorded as soon as the reading was stabilized, and the TEER value calculated as follows.

5 $$TEER = \left( {Ohm2 - Ohm1} \right) \times A$$

where Ohm1 = Resistance of the insert with cell culture medium only, Ohm 2 = Resistance of the insert with cells, A = Surface area of the insert in cm^2^.

The TEER was calculated using the above equation and the result are presented as % of the negative control.

### Statistical analysis and similarity algorithms

All the experiments were repeated three times and data were expressed as mean values ± standard deviation. Data was analyzed using GraphPad Prism 8 (GraphPad Software Inc., La Jolla, CA, USA) and Minitab 18 software (for cellular experiments on intestinal models). In all the cases the one-way analysis of variance (ANOVA) coupled to the Turkeys multiple comparison post hoc test were used to test statistical significance between samples. Differences have been considered significant for p values < 0.05.

Different approaches to assess similarity across the DNs (dissolution and hazard) were used:

o Stochastic likelihood-based BF approach that assesses probabilistically two different properties of the different NFs simultaneously (for more details refer to supplementary information)[26]. This tool was applied on both dissolution and hazard DNs. In brief, for the dissolution DNs, the BF approach was performed comparing either the dissolution % and the half-time values, whereas, for hazard DNs, the comparison was made between the specific biological endpoint addressed (e.g., TEER % data) and the concentration values.
p Clustering approach which estimates groups of NFs based on pairwise distances between all NFs in terms of half-time data (for more details refer to supplementary information)[9].

## Supplementary Information

**Additional file 1. Figure SI1:** GRACIOUS template for generating grouping-based hypotheses and Human oral hypotheses (H-O-) developed for oral ingested NFs. **Table SI1. **Groups by hazard descriptors and following the hazard driven oral hypotheses. **Figure SI2. **Morphology analysis by TEM of Silica NFs dispersed in water (left) and in MEM supplemented with 2mM L-glutamine (right). **Figure SI3. **Size distribution profiles (DH) of Silica NFs dispersed in MilliQ water (Ctrl at t0) and in cell culture medium (NFs at t0 and t24) by DLS analysis. **Table SI2. **DH values of Silica NFs dispersed in MilliQ water (Ctrl at t0) and in cell culture medium (NFs at t0 and t24) by DLS analysis. Table SI3. % of dissolution of the selected silica panel (1 mg/mL) measured after 155 minutes of OGI digestion. **Figure SI4. **Similarity assessment by cluster analysis using the half-time values of OGI dissolution. **Table SI4. **Dissolution rate of the selected silica panel (1 mg) measured in PSF fluid. **Figure SI5. **Similarity assessment by cluster analysis using the half-time values of PSF dissolution. **Figure SI6. **Viability of undifferentiated Caco-2 cells treated with different concentrations of silica NFs, from 0,98 to 125 µg/mL. **Figure SI7. **Impact on barrier integrity of triple intestinal culture model measured daily for 5 days during exposure of (A) 6.72 µg/mL of NFs, (B) 33.6 µg/mL of NFs and (C) 67.2 µg/mL of NFs using TEER. **Figure SI8. **Acellular ROS detection using the DCFH2-DA probe incubated with the tested NFs (final concentrations of 1.56-100 µg/mL). Data are expressed in arbitrary fluorescence units and as mean ± standard deviation (n =3). **Figure SI9. **Representative calibration curve deriving from IL-8 standards dissolved in assay diluent with or without the addition of NFs implemented in the study.

## Data Availability

Data and materials presented in the current study are available from the authors upon request.

## Abbreviations

ALP: Alkaline phosphatase
ANOVA: One-way analysis of variance
ALT: Alanine aminotransferase
AP: Apical
AST: Aspartate aminotransferase
ATCC: American Tissue Culture Collection
BET: Brunauer–Emmett–Teller
BF: Bayes Factor
BL: Basolateral
DCFH.DA: Dichloro-dihydro-fluorescein diacetate
DMEM: Dulbecco’s Modified Eagle’s Medium
DNs: Decision nodes
DLS: Dynamic light scattering
ECACC: European Collection of Authentic Cell Culture
ECHA: European Chemicals Agency
ELISA: Enzyme-linked immunosorbent assays
FBS: Fetal bovine serum
FCS: Fetal calf serum
FDA: Food and Drug Administration
FDA: Fluorescein diacetate
HARN: High aspect ratio nanomaterials
H–O-G: Oral gradual hypothesis
IATA: Integrated Approach to Testing and Assessment
ICP-OES: Inductive coupled plasma optical emission spectrometry
IL-6: Interleukin-6
IL-8: Interleukin-8
LAL: Limulus amebocyte lysate
PSF: Phagolysosomal fluid
LPS: Lipopolysaccharide
MCM: Mesoporous silica nanoparticles
MEM: Minimum essential medium
MWCNT: Multiwalled carbon nanotubes
NEAA: Non-essential amino acid
NMs: Nanomaterials
NFs: Nanoforms
OGI: Oro-gastrointestinal
PBS: Phosphate-buffered saline
PC: Physicochemical
qPCR: Quantitative real-time PCR
RPMI: Roswell Park Memorial Institute
ROS: Reactive oxygen species
SbD: Safe by design
TEM: Transmission electronic microscopy
TEER: Transepithelial electrical resistance
TNF-α: Tumor necrosis factor- alpha

## References

[1] Reach E (2018). Commission Regulation (EU) 2018/1881 of 3 December 2018 Amending Regulation (EC) No 1907/2006 of the European Parliament and of the Council on the Registration, Evaluation, Authorisation and Restriction of Chemicals (REACH) as Regards Annexes I, III, VI, VII, VIII, IX, X, XI, and XII. To Address Nanoforms of Substances. Off J Eur Union.

[2] ECHA (2019) Appendix R.6–1 for Nanoforms Applicable to the Guidance on QSARs and Grouping of Chemicals

[3] Stone V, Gottardo S, Bleeker EA (2020). A framework for grouping and read-across of nanomaterials-supporting innovation and risk assessment. Nano Today.

[4] Murphy FA, Johnston HJ, Dekkers S, et al (2022) How to formulate hypotheses and IATA to support grouping and read-across of nanoforms. ALTEX-Alternatives to animal experimentation 10.14573/altex.2203241 35796348

[5] Murphy F, Dekkers S, Braakhuis H (2021). An integrated approach to testing and assessment of high aspect ratio nanomaterials and its application for grouping based on a common mesothelioma hazard. NanoImpact.

[6] Di Cristo L, Oomen AG, Dekkers S (2021). Grouping hypotheses and an integrated approach to testing and assessment of nanomaterials following oral ingestion. Nanomaterials.

[7] Braakhuis HM, Murphy F, Ma-Hock L (2021). An integrated approach to testing and assessment to support grouping and read-across of nanomaterials after inhalation exposure. Appl Vitro Toxicol.

[8] Di Cristo L, Janer G, Dekkers S (2022). Integrated approaches to testing and assessment for grouping nanomaterials following dermal exposure. Nanotoxicology.

[9] Jeliazkova N, Bleeker E, Cross R (2022). How can we justify grouping of nanoforms for hazard assessment? Concepts and tools to quantify similarity. NanoImpact.

[10] Murugadoss S, Lison D, Godderis L (2017). Toxicology of silica nanoparticles: an update. Arch Toxicol.

[11] Fytianos G, Rahdar A, Kyzas GZ (2020). Nanomaterials in cosmetics: recent updates. Nanomaterials.

[12] Mebert AM, Baglole CJ, Desimone MF, Maysinger D (2017). Nanoengineered silica: properties, applications and toxicity. Food Chem Toxicol.

[13] Wittig A, Gehrke H, Del Favero G (2017). Amorphous silica particles relevant in food industry influence cellular growth and associated signaling pathways in human gastric carcinoma cells. Nanomaterials.

[14] Younes M, Aggett P, Aguilar F, EFSA Panel on Food Additives and Nutrient Sources added to Food (ANS) (2018). Re-evaluation of silicon dioxide (E 551) as a food additive. EFSA J.

[15] Ang CW, Tan L, Qu Z (2021). Mesoporous silica nanoparticles improve oral delivery of antitubercular bicyclic nitroimidazoles. ACS Biomater Sci Eng.

[16] Florek J, Caillard R, Kleitz F (2017). Evaluation of mesoporous silica nanoparticles for oral drug delivery–current status and perspective of MSNs drug carriers. Nanoscale.

[17] Sohal IS, Cho YK, O’Fallon KS (2018). Dissolution behavior and biodurability of ingested engineered nanomaterials in the gastrointestinal environment. ACS Nano.

[18] Keller JG, Persson M, Mueller P (2021). Variation in dissolution behavior among different nanoforms and its implication for grouping approaches in inhalation toxicity. NanoImpact.

[19] Rasmussen K, Mech A, Mast J, et al (2013) Synthetic amorphous silicon dioxide (NM-200, NM-201, NM-202, NM-203, NM-204): characterisation and physico-chemical properties. JRC Scientific and Policy Reports

[20] Catalano F, Pompa PP (2019). Design rules for mesoporous silica toward the nanosize: a systematic study. ACS Appl Mater Interfaces.

[21] DeLoid G, Cohen JM, Darrah T (2014). Estimating the effective density of engineered nanomaterials for in vitro dosimetry. Nat Commun.

[22] Bove P, Malvindi MA, Kote SS (2017). Dissolution test for risk assessment of nanoparticles: a pilot study. Nanoscale.

[23] Carnovale C, Guarnieri D, Di Cristo L (2021). Biotransformation of silver nanoparticles into oro-gastrointestinal tract by integrated in vitro testing assay: generation of exposure-dependent physical descriptors for nanomaterial grouping. Nanomaterials.

[24] Guarnieri D, Sánchez-Moreno P, Del Rio Castillo AE (2018). Biotransformation and biological interaction of graphene and graphene oxide during simulated oral ingestion. Small.

[25] Committee ES (2021) Guidance on risk assessment of nanomaterials to be applied in the food and feed chain: human and animal health 10.2903/j.efsa.2021.6768 PMC8331059 34377190

[26] Tsiliki G, Seleci DA, Zabeo A (2022). Bayesian based similarity assessment of nanomaterials to inform grouping. NanoImpact.

[27] Ude VC, Brown DM, Viale L (2017). Impact of copper oxide nanomaterials on differentiated and undifferentiated Caco-2 intestinal epithelial cells; assessment of cytotoxicity, barrier integrity, cytokine production and nanomaterial penetration. Part Fibre Toxicol.

[28] Ude VC, Brown DM, Stone V, Johnston HJ (2019). Using 3D gastrointestinal tract in vitro models with microfold cells and mucus secreting ability to assess the hazard of copper oxide nanomaterials. J Nanobiotechnol.

[29] Farcal L, Torres Andón F, Di Cristo L (2015). Comprehensive in vitro toxicity testing of a panel of representative oxide nanomaterials: first steps towards an intelligent testing strategy. PLoS ONE.

[30] Drasler B, Sayre P, Steinhäuser KG (2017). In vitro approaches to assess the hazard of nanomaterials. NanoImpact.

[31] Croissant JG, Butler KS, Zink JI, Brinker CJ (2020). Synthetic amorphous silica nanoparticles: toxicity, biomedical and environmental implications. Nat Rev Mater.

[32] Lison D, Thomassen LC, Rabolli V (2008). Nominal and effective dosimetry of silica nanoparticles in cytotoxicity assays. Toxicol Sci.

[33] Sousa de Almeida M, Taladriz-Blanco P, Drasler B (2022). Cellular uptake of silica and gold nanoparticles induces early activation of nuclear receptor NR4A1. Nanomaterials.

[34] Liu C, Chu D, Kalantar-Zadeh K (2021). Cytokines: from clinical significance to quantification. Adv Sci.

[35] Ameh T, Sayes CM (2019). The potential exposure and hazards of copper nanoparticles: a review. Environ Toxicol Pharmacol.

[36] Tassinari R, Di Felice G, Butteroni C (2020). Hazard identification of pyrogenic synthetic amorphous silica (NM-203) after sub-chronic oral exposure in rat: a multitarget approach. Food Chem Toxicol.

[37] Tarantini A, Huet S, Jarry G (2015). Genotoxicity of synthetic amorphous silica nanoparticles in rats following short-term exposure. Part 1: Oral route. Environ Mol Mutagen.

[38] De Jong WH, De Rijk E, Bonetto A (2019). Toxicity of copper oxide and basic copper carbonate nanoparticles after short-term oral exposure in rats. Nanotoxicology.

[39] Buesen R, Landsiedel R, Sauer UG (2014). Effects of SiO2, ZrO2, and BaSO4 nanomaterials with or without surface functionalization upon 28-day oral exposure to rats. Arch Toxicol.

[40] Cabellos J, Gimeno-Benito I, Catalán J (2020). Short-term oral administration of non-porous and mesoporous silica did not induce local or systemic toxicity in mice. Nanotoxicology.

[41] Boudard D, Aureli F, Laurent B (2019). Chronic oral exposure to synthetic amorphous silica (NM-200) results in renal and liver lesions in mice. Kidney Int Rep.

[42] Tassinari R, Martinelli A, Valeri M, Maranghi F (2021). Amorphous silica nanoparticles induced spleen and liver toxicity after acute intravenous exposure in male and female rats. Toxicol Ind Health.

[43] Deng Y-D, Zhang X-D, Yang X-S (2021). Subacute toxicity of mesoporous silica nanoparticles to the intestinal tract and the underlying mechanism. J Hazard Mater.

[44] Mohammadpour R, Cheney DL, Grunberger JW (2020). One-year chronic toxicity evaluation of single dose intravenously administered silica nanoparticles in mice and their Ex vivo human hemocompatibility. J Control Release.

[45] Villani P, Eleuteri P, Pacchierotti F (2022). Pyrogenic synthetic amorphous silica (NM-203): Genotoxicity in rats following sub-chronic oral exposure. Mutat Res Genet Toxicol Environ Mutagen.

[46] Tassinari R, Cordelli E, Eleuteri P (2021). Effects of sub-chronic oral exposure to pyrogenic synthetic amorphous silica (NM-203) in male and female Sprague-Dawley rats: focus on reproductive systems. Reprod Toxicol.

[47] Dekkers S, Krystek P, Peters RJ (2011). Presence and risks of nanosilica in food products. Nanotoxicology.

[48] Yao Y, Zang Y, Qu J (2019). The toxicity of metallic nanoparticles on liver: the subcellular damages, mechanisms, and outcomes. Int J Nanomed.

[49] Boey A, Ho HK (2020). All roads lead to the liver: metal nanoparticles and their implications for liver health. Small.

[50] Kermanizadeh A, Gaiser BK, Hutchison GR, Stone V (2012). An in vitro liver model-assessing oxidative stress and genotoxicity following exposure of hepatocytes to a panel of engineered nanomaterials. Part Fibre Toxicol.

[51] Sahu SC, Zheng J, Graham L (2014). Comparative cytotoxicity of nanosilver in human liver HepG2 and colon Caco2 cells in culture. J Appl Toxicol.

[52] Yu H, Huang Q (2013). Investigation of the cytotoxicity of food-grade nanoemulsions in Caco-2 cell monolayers and HepG2 cells. Food Chem.

[53] Brand W, van Kesteren PC, Peters RJ, Oomen AG (2021). Issues currently complicating the risk assessment of synthetic amorphous silica (SAS) nanoparticles after oral exposure. Nanotoxicology.

[54] Kermanizadeh A, Valli J, Sanchez K (2022). Particulate and drug-induced toxicity assessed in novel quadruple cell human primary hepatic disease models of steatosis and pre-fibrotic NASH. Arch Toxicol.

[55] Jeliazkova N, Ma-Hock L, Janer G (2022). Possibilities to group nanomaterials across different substances–a case study on organic pigments. NanoImpact.

[56] Murphy F, Jacobsen NR, Di Ianni E (2022). Grouping MWCNTs based on their similar potential to cause pulmonary hazard after inhalation: a case-study. Part Fibre Toxicol.

[57] Di Cristo L, Mc Carthy S, Paton K (2018). Interplay between oxidative stress and endoplasmic reticulum stress mediated-autophagy in unfunctionalised few-layer graphene-exposed macrophages. 2D Mater.

[58] Di Cristo L, Maguire CM, Mc Quillan K (2018). Towards the identification of an in vitro tool for assessing the biological behavior of aerosol supplied nanomaterials. Int J Environ Res Public Health.

[59] FDA U (2018) Guidance for industry—pyrogen and endotoxins testing. Services USDoHH (Eds.) US Food and Drug Administration, Silver Spring 11

[60] Keller JG, Peijnenburg W, Werle K (2020). Understanding dissolution rates via continuous flow systems with physiologically relevant metal ion saturation in lysosome. Nanomaterials.

[61] Guldberg M, Christensen VR, Perander M (1998). Measurement of in-vitro fibre dissolution rate at acidic pH. Ann Occup Hyg.

[62] OECD (2020) Guidance document for the testing of dissolution and dispersion stability of nanomaterials and the use of the data for further environmental testing and assessment strategies

[63] Stefaniak AB, Guilmette RA, Day GA (2005). Characterization of phagolysosomal simulant fluid for study of beryllium aerosol particle dissolution. Toxicol In Vitro.

[64] Behrens I, Stenberg P, Artursson P, Kissel T (2001). Transport of lipophilic drug molecules in a new mucus-secreting cell culture model based on HT29-MTX cells. Pharm Res.

[65] Pulvertaft RJV (1964). Cytology of Burkitt’s tumour African lymphoma. Lancet.

[66] Schimpel C, Teubl B, Absenger M (2014). Development of an advanced intestinal in vitro triple culture permeability model to study transport of nanoparticles. Mol Pharm.

[67] Rotoli BM, Bussolati O, Costa AL (2012). Comparative effects of metal oxide nanoparticles on human airway epithelial cells and macrophages. J Nanopart Res.

[68] Prozeller D, Morsbach S, Landfester K (2019). Isothermal titration calorimetry as a complementary method for investigating nanoparticle–protein interactions. Nanoscale.

[69] Maiorano G, Sabella S, Sorce B (2010). Effects of cell culture media on the dynamic formation of protein- nanoparticle complexes and influence on the cellular response. ACS Nano.

[70] Chomczynski P (1993). A reagent for the single-step simultaneous isolation of RNA, DNA and proteins from cell and tissue samples. Biotechniques.

[71] Wilfinger WW, Mackey K, Chomczynski P (1997). Effect of pH and ionic strength on the spectrophotometric assessment of nucleic acid purity. Biotechniques.

[72] Pfaffl MW (2001). A new mathematical model for relative quantification in real-time RT–PCR. Nucleic Acids Res.

