# Supplementary material for: Grouping of orally ingested silica nanomaterials via use of an integrated approach to testing and assessment to streamline risk assessment
Source: Part Fibre Toxicol. 2022 Dec 2;19:68. doi: 10.1186/s12989-022-00508-4 (PMC9719179; doi:10.1186/s12989-022-00508-4)
Supplement: Supplementary file 1 — Additional file 1. Figure SI1: GRACIOUS template for generating grouping-based hypotheses and Human oral hypotheses (H-O-) developed for oral ingested NFs. Table SI1. Groups by hazard descriptors and following the hazard driven oral hypotheses. Figure SI2. Morphology analysis by TEM of Silica NFs dispersed in water (left) and in MEM supplemented with 2mM L-glutamine (right). Figure SI3. Size distribution profiles (DH) of Silica NFs dispersed in MilliQ water (Ctrl at t0) and in cell culture medium (NFs at t0 and t24) by DLS analysis. Table SI2. DH values of Silica NFs dispersed in MilliQ water (Ctrl at t0) and in cell culture medium (NFs at t0 and t24) by DLS analysis. Table SI3. % of dissolution of the selected silica panel (1 mg/mL) measured after 155 minutes of OGI digestion. Figure SI4. Similarity assessment by cluster analysis using the half-time values of OGI dissolution. Table SI4. Dissolution rate of the selected silica panel (1 mg) measured in PSF fluid. Figure SI5. Similarity assessment by cluster analysis using the half-time values of PSF dissolution. Figure SI6. Viability of undifferentiated Caco-2 cells treated with different concentrations of silica NFs, from 0,98 to 125 µg/mL. Figure SI7. Impact on barrier integrity of triple intestinal culture model measured daily for 5 days during exposure of (A) 6.72 µg/mL of NFs, (B) 33.6 µg/mL of NFs and (C) 67.2 µg/mL of NFs using TEER. Figure SI8. Acellular ROS detection using the DCFH2-DA probe incubated with the tested NFs (final concentrations of 1.56-100 µg/mL). Data are expressed in arbitrary fluorescence units and as mean ± standard deviation (n =3). Figure SI9. Representative calibration curve deriving from IL-8 standards dissolved in assay diluent with or without the addition of NFs implemented in the study. [file 12989_2022_508_MOESM1_ESM.docx]

**Grouping of orally ingested silica nanomaterials *via* use of an Integrated Approach to Testing and Assessment to streamline risk assessment**

Luisana Di Cristo^1*^, Victor C. Ude^2^, Georgia Tsiliki^3^, Giuseppina Tatulli^4^, Alessio Romaldini^1^ Fiona Murphy^2^, Wendel Wohlleben^5^, Agnes G. Oomen^6, 7^, Pier Paolo Pompa^4^, Josje Arts^8^, Vicki Stone^2^ and Stefania Sabella^1*^

^1^ D3 PharmaChemistry, Nanoregulatory Group, Italian Institute of Technology, Via Morego, 30 – 16163 Genova, Italy

^2^Nano Safety Research Group, School of Engineering and Physical Sciences, Heriot Watt University, Edinburgh, EH14 4AS, United Kingdom

^3^Institute for the Management of Information Systems, Athena Research Center, Marousi, Greece

^4^ Nanobiointeractions & Nanodiagnostics, Istituto Italiano di Tecnologia (IIT), Via Morego, 30 – 16163 Genova, Italy

^5^BASF SE, Dept. Material Physics and Dept of Experimental Toxicology & Ecology, Ludwigshafen, Germany

^6^National Institute for Public Health and the Environment (RIVM), Bilthoven, The Netherlands

^7^ University of Amsterdam, Institute for Biodiversity and Ecosystem Dynamics, Amsterdam, The Netherlands

^8^ Nouryon, Arnhem, the Netherlands

*: corresponding authors

**Supporting information**


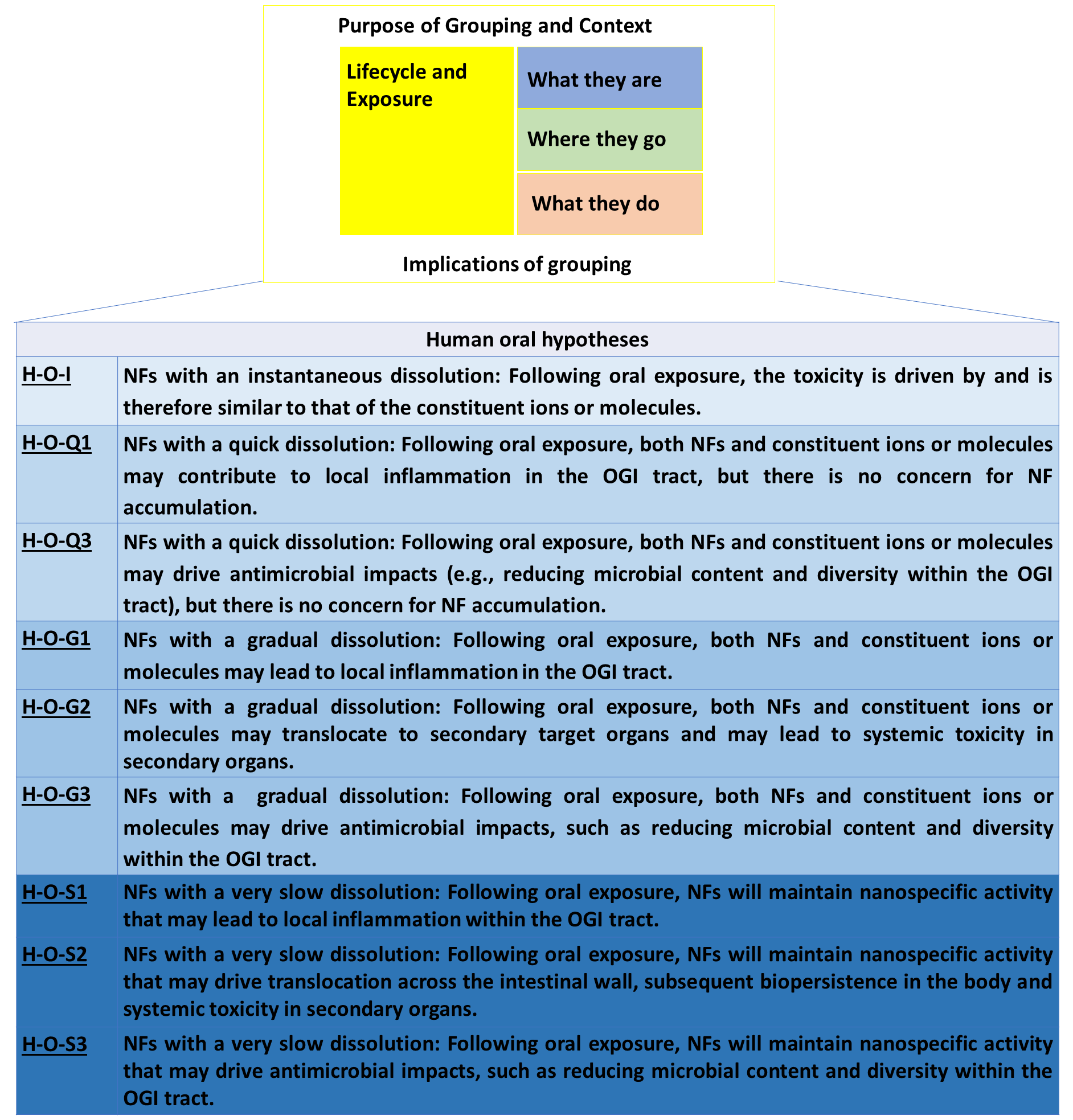


**Figure SI1**. A: GRACIOUS template for generating grouping-based hypotheses [1, 2] and (B) Human oral hypotheses (H-O-) developed for oral ingested NFs. (Di Cristo et al. © MDPI, 2021)


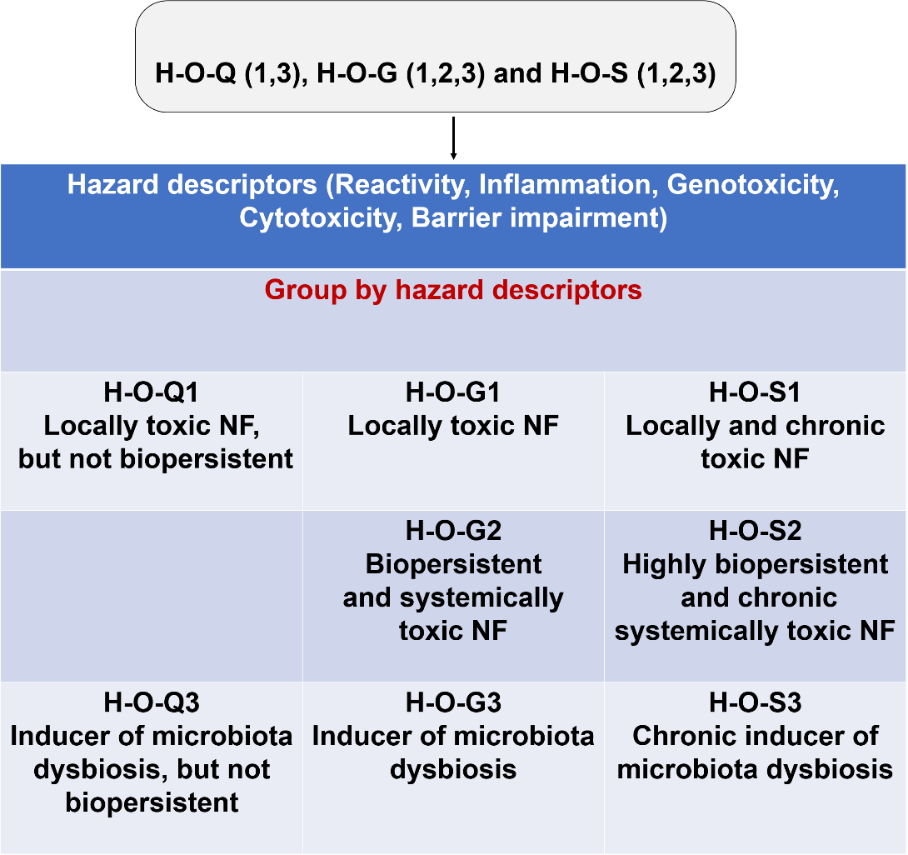


**Table SI1**. Groups by hazard descriptors and following the hazard driven oral hypotheses (Di Cristo et al. © MDPI, 2021)


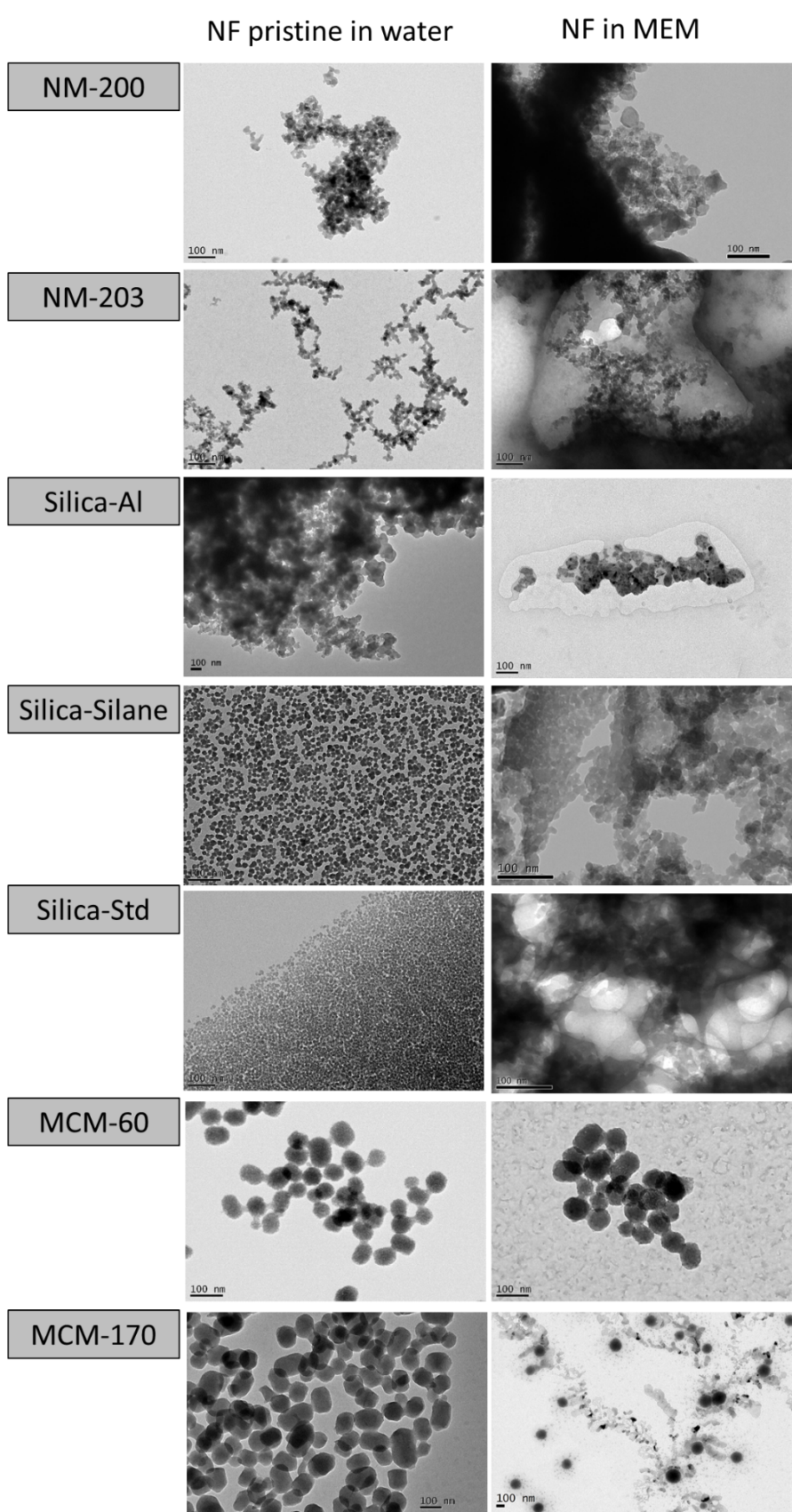


**Figure SI2**. Morphology analysis by TEM of Silica NFs dispersed in water (left) and in MEM supplemented with 2mM L-glutamine (right).


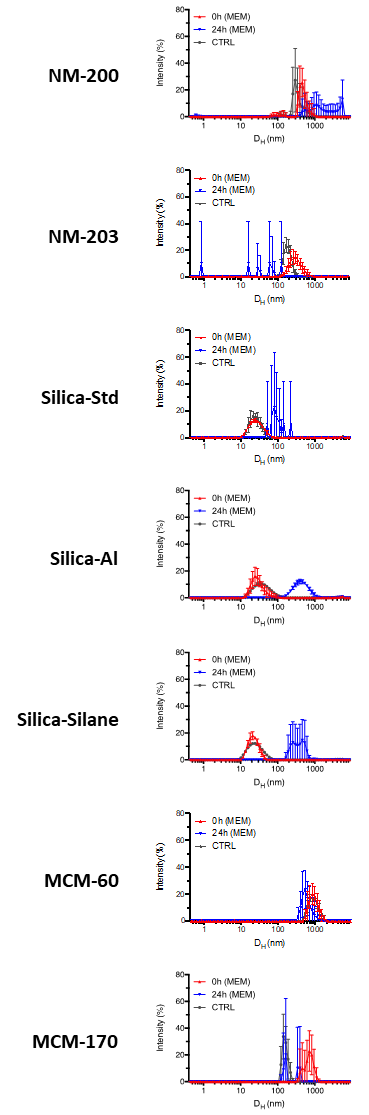


**Figure SI3**. Size distribution profiles (D_H_) of Silica NFs dispersed in MilliQ water (Ctrl at t_0_) and in cell culture medium (NFs at t_0_ and t_24_) by DLS analysis.

|  | Hydrodynamic diameter (D_H_) ± SD  Ctrl (NF in water, t_0_) | Hydrodynamic diameter (D_H_) ± SD  NF in MEM (t_0_) | Hydrodynamic diameter (D_H_) ± SD  NF in MEM (t24) |
| --- | --- | --- | --- |
| NM-200 | 383.19± 136.08 | 504.44±119.63 | 2353.71±1721.39 |
| NM-203 | 192.40± 24.63 | 314.42±46.60 | 11.47±16.04 |
| Silica-Std | 25.67± 2.89 | 27.72±1.88 | 100.98±48.96 |
| Silica-Silane | 26.74± 1.19 | 23.18±1.65 | 368.08±125.31 |
| Silica-Al | 42.66± 4.79 | 31.50±7.89 | 459.99±29.46 |
| MCM-60 | 929.82± 102.10 | 928.62±142.03 | 593.03±167.77 |
| MCM-170 | 160.26 ± 17.66 | 702.35±165.32 | 120.65±149.82 |

**Table SI2**. D_H_ values of Silica NFs dispersed in MilliQ water (Ctrl at t_0_) and in cell culture medium (NFs at t_0_ and t_24_) by DLS analysis.

**Table SI3.** % of dissolution of the selected silica panel (1 mg/mL) measured after 155 minutes of OGI digestion.

|  | NM-200 | NM-203 | Silica-Std | Silica-Al | Silica-Silane | MCM-60 | MCM-170 |
| --- | --- | --- | --- | --- | --- | --- | --- |
| % of dissolution  (mean ± standard deviation) | 6.85±3.37 | 5.66±2.62 | 7.87±3.30 | 6.53±2.32 | 4.34±2.52 | 23.47±9.21 | 18.41±2.17 |

**
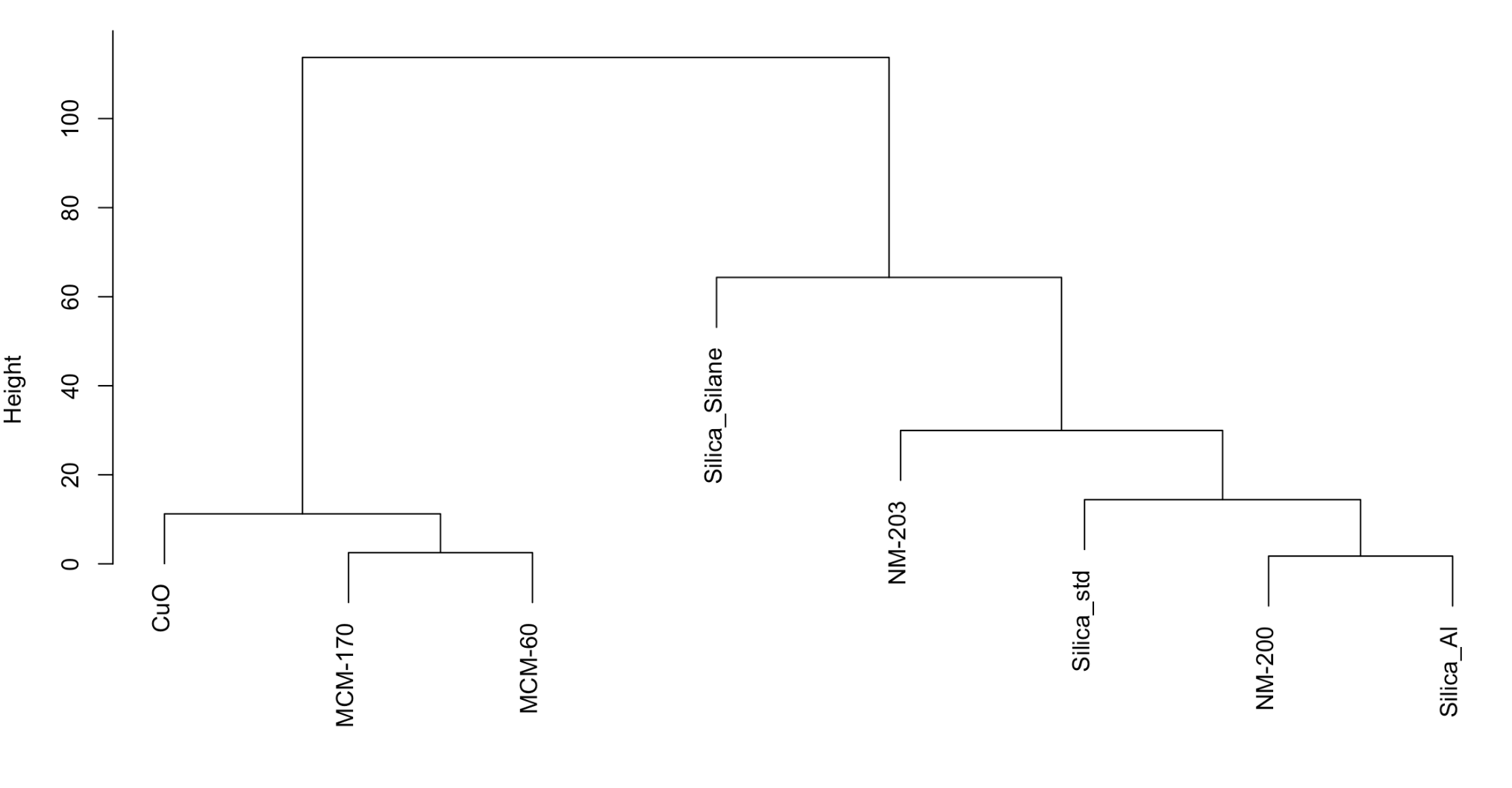
**

**Figure SI4.** Similarity assessment by cluster analysis using the half-time values of OGI dissolution.

**Table SI4.** Dissolution k rate of the selected silica panel (1 mg) measured in PSF fluid (Keller et al., 2021).

|  | NM-200 | NM-203 | Silica-Std | Silica-Al | Silica-Silane | MCM-60 | MCM-170 |
| --- | --- | --- | --- | --- | --- | --- | --- |
| Dissolution rate (ng/cm^2^/h) | 0.58 | 0.40 | 3.59 | 3.98 | 3.76 | 0.159 | 0.348 |


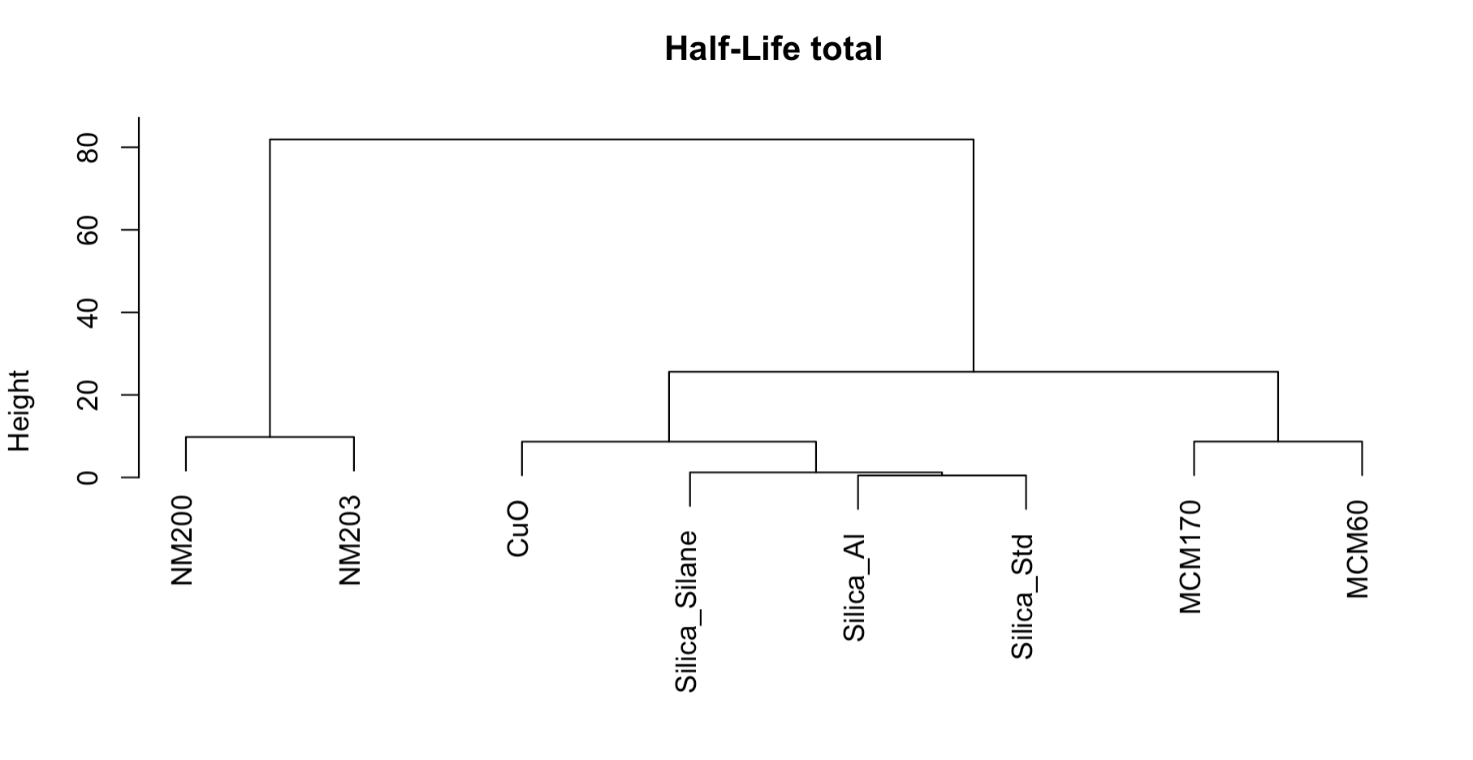


**Figure SI5.** Similarity assessment by cluster analysis using the half-time values of PSF dissolution.

**Figure SI6.** Viability of undifferentiated Caco-2 cells treated with different concentrations of silica NFs, from 0,98 to 125 µg/mL.


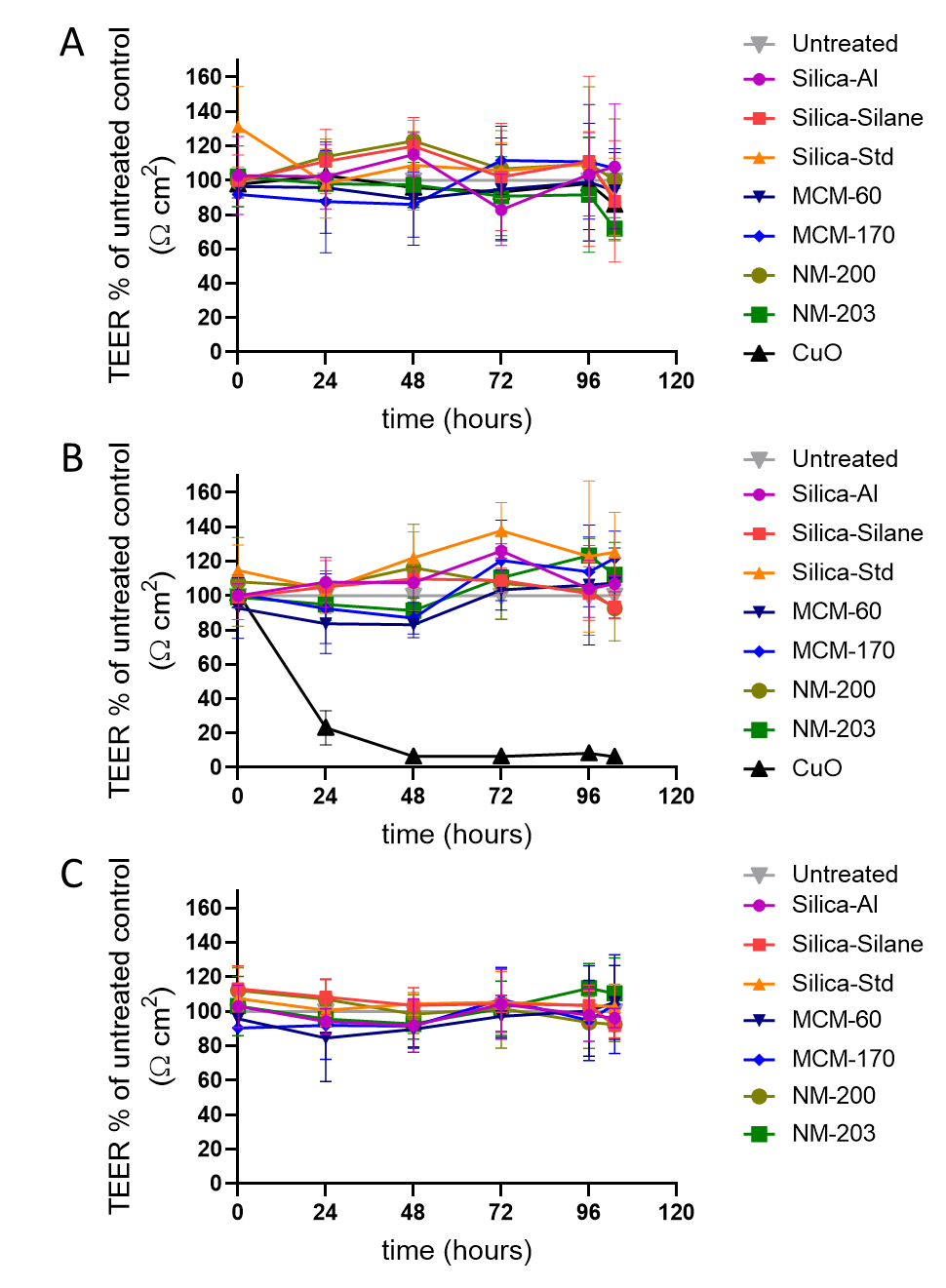


**Figure SI7**. Impact on barrier integrity of triple intestinal culture model measured daily for 5 days during exposure of (A) 6.72 µg/mL of NFs, (B) 33.6 µg/mL of NFs and (C) 67.2 µg/mL of NFs using TEER.

**Figure SI8.** Acellular ROS detection using the DCFH_2_-DA probe incubated with the tested NFs (final concentrations of 1.56-100 µg/mL). Data are expressed in arbitrary fluorescence units and as mean ± standard deviation (n =3).

**Figure SI9.** Representative calibration curve deriving from IL-8 standards dissolved in assay diluent with or without the addition of NFs implemented in the study.

**Stochastic likelihood-based BF approach**

This method is introduced by Tsiliki et al. [3] and it has been applied to reactivity data as explained in Ag Seleci et al.[4]. In brief, the pairwise similarity assessment examined whether any two selected NFs are derived from the same log-normal distribution (with the same mean and variance parameters) as opposed to completely different distributions. Likelihood-based calculations for the two assumptions above are compared via Bayes Factors (BF), which are then rescaled to a scalar value in the range between 0 and 1, with values close to 1 denoting highly similar NFs.

**Clustering approach**

Similarities in dissolution were also quantified per single descriptor (half-time values) using hierarchical clustering with the Manhattan distance metric. The Manhattan distances for the dissolution descriptor data were used as an adjustment to the BF calculations in order to address large discrepancies between NFs values. The later approach is a multi-component analysis, which is estimating existing groups for all Silica based NFs considered here[5].

**Concentration calculation**

The concentration of NFs exposed to the *in vitro* intestinal models were calculated based on published estimations of adult daily intake of SiO_2_ NMs, length and diameter of human small intestine. The average daily intake of SiO_2_ NMs has been reported to be approximately 35 mg [6]. The average length and diameter of the small intestine are 459.6 cm and 3 cm respectively [7]. We made the assumption to not consider the surface area amplification due to the villi and microvilli. Therefore, the surface area of the intestine is:

SA = L×D×π

Where L= length of the intestine, D = diameter of the intestine and π=3.14

SA= 459.6×3×3.14 = 4,329.432 cm^2^.

To calculate exposure concentration per square centimeter, the daily SiO_2_ ingestion was divided by surface area of the intestine without villi and microvilli amplification, 35 mg/4,329.432 cm^2^ = 8.1×10^-3^ mg/cm^2^ = 8.1 µg/cm^2^. Based on this, 3, 15 and 30 µg/cm^2^ corresponding to 6.72, 33.6 and 67.2 µg/mL according to the cell culture system used, were the selected doses for exposure the 3D *in vitro* intestinal model to cover a worst-case scenario.

**References**

1. Stone V, Gottardo S, Bleeker EA, et al (2020) A framework for grouping and read-across of nanomaterials-supporting innovation and risk assessment. Nano Today 35:100941

2. Murphy FA, Johnston HJ, Dekkers S, et al (2022) How to formulate hypotheses and IATA to support grouping and read-across of nanoforms. ALTEX-Alternatives to animal experimentation

3. Tsiliki G, Seleci DA, Zabeo A, et al (2022) Bayesian based similarity assessment of nanomaterials to inform grouping. NanoImpact 25:100389

4. Seleci DA, Tsiliki G, Werle K, et al (2022) Determining nanoform similarity via assessment of surface reactivity by abiotic and In vitro assays. NanoImpact 26:100390

5. Jeliazkova N, Bleeker E, Cross R, et al (2022) How can we justify grouping of nanoforms for hazard assessment? Concepts and tools to quantify similarity. NanoImpact 25:100366

6. Lomer MC, Hutchinson C, Volkert S, et al (2004) Dietary sources of inorganic microparticles and their intake in healthy subjects and patients with Crohn’s disease. British Journal of Nutrition 92:947–955

7. Hosseinpour M, Behdad A (2008) Evaluation of small bowel measurement in alive patients. Surgical and radiologic anatomy 30:653–655
